# Machine learning in epidemiology: Neural networks forecasting of monkeypox cases

**DOI:** 10.1371/journal.pone.0300216

**Published:** 2024-05-01

**Authors:** Lulah Alnaji

**Affiliations:** Department of Mathematics, University of Hafr Al-Batin, Hafr Al-Batin, Saudi Arabia; Hosei University: Hosei Daigaku, JAPAN

## Abstract

This study integrates advanced machine learning techniques, namely Artificial Neural Networks, Long Short-Term Memory, and Gated Recurrent Unit models, to forecast monkeypox outbreaks in Canada, Spain, the USA, and Portugal. The research focuses on the effectiveness of these models in predicting the spread and severity of cases using data from June 3 to December 31, 2022, and evaluates them against test data from January 1 to February 7, 2023. The study highlights the potential of neural networks in epidemiology, especially concerning recent monkeypox outbreaks. It provides a comparative analysis of the models, emphasizing their capabilities in public health strategies. The research identifies optimal model configurations and underscores the efficiency of the Levenberg-Marquardt algorithm in training. The findings suggest that ANN models, particularly those with optimized Root Mean Squared Error, Mean Absolute Percentage Error, and the Coefficient of Determination values, are effective in infectious disease forecasting and can significantly enhance public health responses.

## Introduction

The Monkeypox Virus (MPXV), a member of the Orthopoxvirus genus, is the causative agent of the infectious disease known as monkeypox. This virus is predominantly found in Central and West African countries, with sporadic cases reported in other regions, including the United States and the United Kingdom [1–3].

Transmission of MPXV to humans often occurs through direct contact with infected animals or contaminated objects, such as body fluids, sores, or bedding [1, 2, 4]. Human-to-human transmission is also possible, mainly through close physical interaction with infected individuals or exposure to their bodily fluids [1, 4]. Symptoms of MPXV infection include fever, headache, muscle aches, and a characteristic rash that spreads across the body [1, 4]. In severe cases, complications such as pneumonia, sepsis, and encephalitis can occur [1, 2, 4].

No specific antiviral treatment for MPXV currently exists; however, supportive care can aid in symptom management and reduction of complication risks [1, 2, 4]. Vaccination against smallpox has shown some effectiveness in preventing monkeypox, but routine smallpox immunization is no longer practiced [1, 2]. Therefore, public health measures such as contact tracing, quarantine, and isolation are essential in controlling the spread of the disease [1, 2, 4].

MPXV, part of the Orthopoxvirus family, was first identified in monkeys in the Democratic Republic of the Congo in 1958 and in humans in 1970. The virus is endemic in certain areas of Central and West Africa, with occasional outbreaks. Reports of cases in countries outside Africa, including the United States, Canada, Portugal, and Spain, have increased recently [5].

The first recorded case of MPXV in the United States occurred in 2003 in a traveler from West Africa. This led to an investigation that identified 47 confirmed or probable cases across six states, primarily linked to prairie dogs infected with the virus. In Canada, a similar outbreak occurred in 2003, with two confirmed cases of MPXV in individuals who had traveled to West Africa [6, 7].

Portugal reported its first outbreak of MPXV in 2018, with nine cases linked to recent travel to Nigeria. In 2021, Spain experienced its first outbreak of MPXV, with two cases also related to travel to Nigeria. These instances highlight the increasing frequency of MPXV cases outside Africa, underscoring the need for vigilant surveillance and preparedness to manage any outbreaks [8].

The utilization of machine learning in epidemiological research represents a transformative approach to understanding and managing infectious diseases. Building on the existing state of the art in disease forecasting, particularly leveraging machine learning techniques, our study aims to enhance the predictive modeling of monkeypox spread. While previous studies like [9] have developed neural network models for forecasting monkeypox in various countries, our research focuses on employing Artificial Neural Networks (ANN), Long Short-Term Memory (LSTM), and Gated Recurrent Unit (GRU) models to predict monkeypox cases in Canada, Spain, the USA, and Portugal. This approach not only addresses a gap in monkeypox research but also compares the efficacy of different neural network models, contributing perspective to the field.

The recent upsurge in monkeypox cases globally highlights the need for improved disease monitoring and forecasting methods. In this context, our study introduces advanced machine learning techniques, namely ANN, LSTM, and GRU models, to predict monkeypox outbreaks. This research is significant in its focus on the latest MPXV outbreaks and its comparative evaluation of different neural network models for forecasting the disease’s spread in various countries.

## Literature review

The study of infectious diseases, particularly emerging viruses like MPXV, has increasingly incorporated machine learning approaches to enhance prediction and management strategies. Key studies in this field have demonstrated the utility of various neural network models, such as ANN, LSTM, and GRU, in understanding and forecasting disease patterns [10–15]. Our work builds upon these foundations, particularly focusing on recent developments in monkeypox forecasting.

Early detection and prediction of infectious diseases like MPXV are crucial for effective management and response. ANN approaches, as utilized in forecasting COVID-19 cases in Pakistan, provide valuable insights for healthcare professionals and policymakers [16].

ANN techniques are increasingly being used to predict patient outcomes in various diseases, including COVID-19, breast cancer, and cardiovascular disease. For example, ANN models were employed in assessing breast cancer risk among Iranian women [17].

While prior research like [9] has leveraged neural networks for predicting MPXV spread in specific regions, our study extends this application to Canada, Spain, the USA, and Portugal. This expansion is crucial, given the distinct epidemiological profiles and healthcare systems in these countries. Such comparative analysis contributes novel insights into the geographical variance in MPXV outbreak dynamics.

The role of machine learning in epidemiological modeling has evolved rapidly, with recent advances highlighting its potential in real-time disease surveillance and response planning. Studies have explored various machine learning techniques, including deep learning and predictive analytics, to enhance the accuracy of disease outbreak predictions and to understand transmission dynamics [16, 18–20].

Our study contributes to this growing body of literature by employing a combination of ANN, LSTM, and GRU models, enhanced with the ADAM optimizer [21] and the Levenberg-Marquardt learning algorithm [22]. This approach not only allows for a comprehensive analysis of MPXV spread but also offers a methodological framework that can be adapted for other infectious diseases. The integration of advanced machine learning models in our research addresses a critical gap in current epidemiological studies.

**Fig 1:**
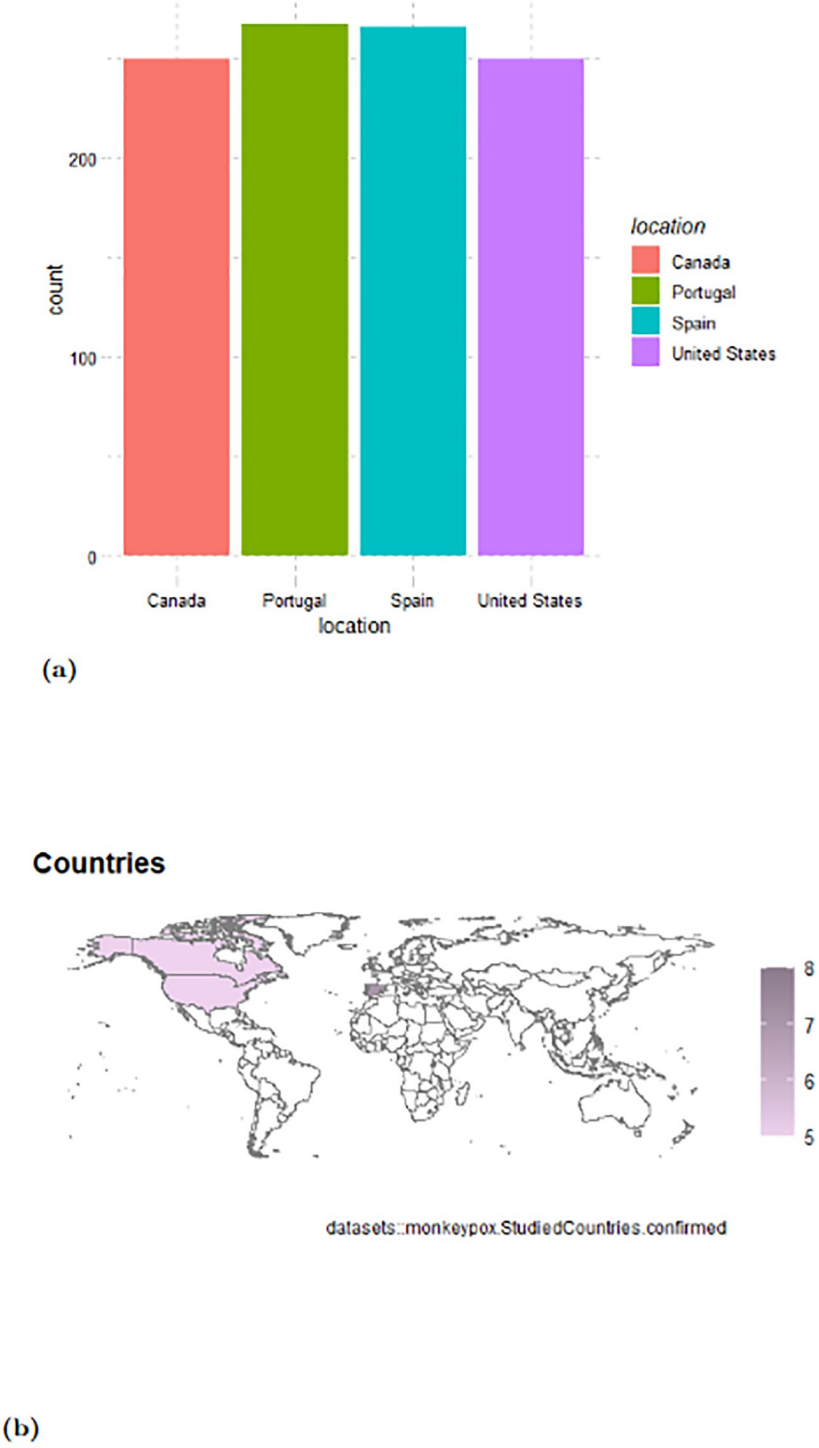
(a) Distribution of confirmed Monkeypox cases across the studied nations, (b) Geographical representation of the studied nations on a global map.

**Fig 2:**
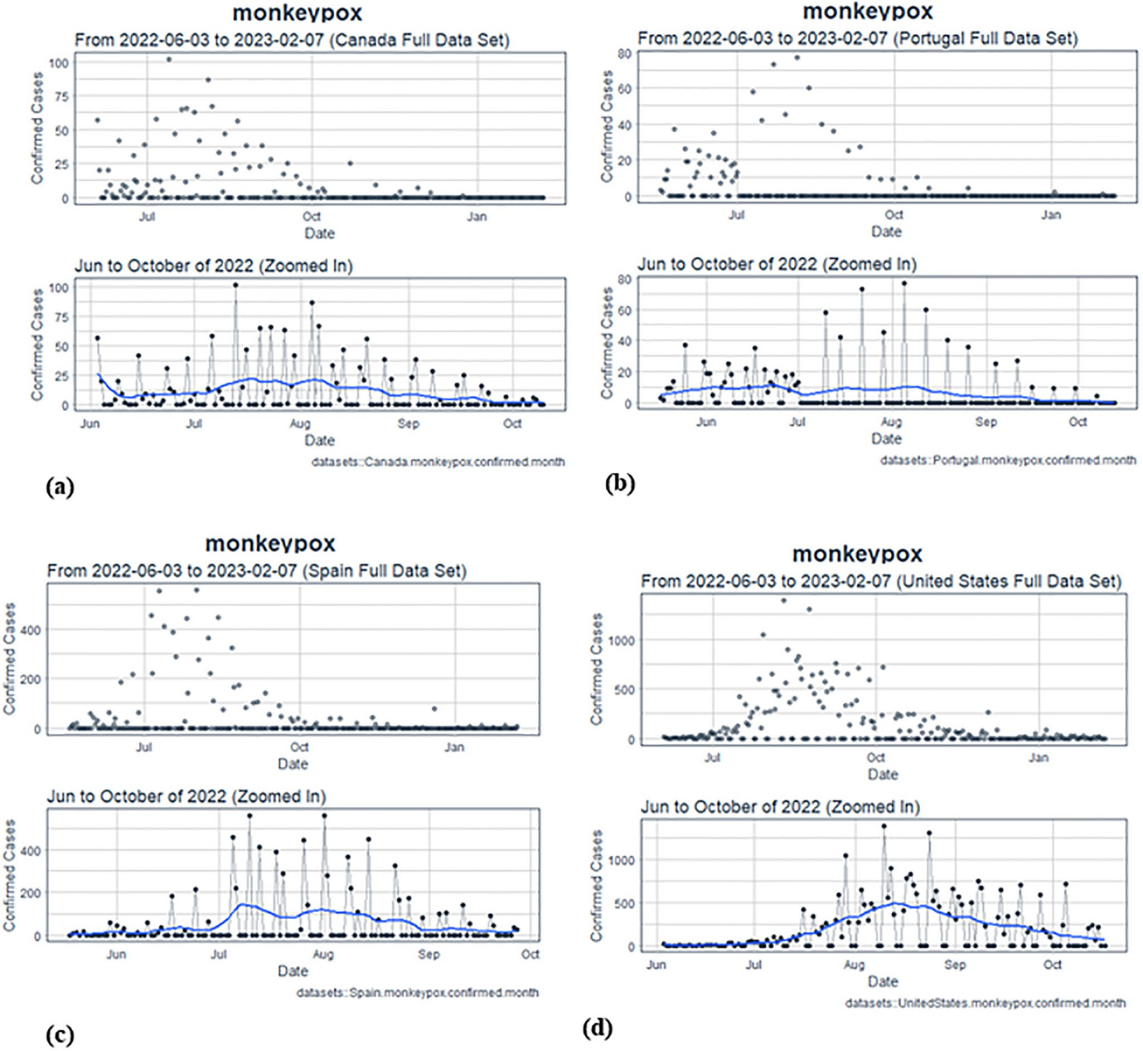
(a) Sequence of confirmed MPXV instances in Canada, with a detailed view of the peak interval (June to October 2022), (b) Sequence of confirmed MPXV instances in Portugal, with a detailed view of the peak interval (June to October 2022), (c) Sequence of confirmed MPXV instances in Spain, with a detailed view of the peak interval (June to October 2022), (d) Sequence of confirmed MPXV instances in the USA, with a detailed view of the peak interval (June to October 2022).

The study utilizes a range of ANN models, including LSTM and GRU, to predict MPXV cases in the USA, Canada, Spain, and Portugal, based on existing datasets. The comparative analysis of these countries will assist healthcare authorities in formulating appropriate response strategies. This research is the first in-depth study using ANN on recent MPXV outbreaks, offering new insights into the epidemic’s dynamics. Time series dataset of MPXV cases from each country, along with statistical graphs of confirmed cases, is presented [23]. The distribution and geographical representation of confirmed Monkeypox cases across the studied nations are depicted in Figs 1 and 2 (Fig 1a shows the distribution of confirmed cases, while Fig 1b provides a geographical representation on a global map). Additionally, the sequence of confirmed MPXV instances, detailed with peak intervals from June to October 2022 for Canada, Portugal, Spain, and the USA, are illustrated in Fig 2a–2d.

The prediction model uses data from the “Our World in Data” website, employing neural network, LSTM, and GRU models. The model’s performance is enhanced using an Adaptive Moment Estimation (ADAM) optimizer [21]. Additionally, a Levenberg-Marquardt (LM) learning algorithm is implemented for a single hidden layer ANN model, optimizing the number of neurons using the K-fold cross-validation early stopping validation approach [22]. ANN-based regression models have been effective in predicting the spread of infectious diseases like MPXV. These models enable informed decision-making by healthcare professionals and policymakers in controlling disease spread and responding effectively to outbreaks. ANN models have been applied in various domains for time-series prediction, demonstrating their versatility and efficacy [10–15].

The remainder of this paper is organized as follows: The Methodology section discusses the methodology used in this study. The Results and Discussions section presents the findings of the research. Following that, the Forecasting Methodology section covers the approach taken for forecasting. The paper concludes with the Conclusion section, summarizing the study’s key findings.

## Methodology

In the manuscript, the choice of modeling methods, including ANN, LSTM, and GRU, is justified by their proven effectiveness in time-series analysis and epidemiological forecasting. ANN is renowned for its ability to model complex nonlinear relationships, making it ideal for predicting disease spread [24]. LSTM and GRU, as advanced recurrent neural networks, effectively capture temporal dependencies in data, crucial for accurate disease trend predictions [25–27]. These methodologies are selected for their ability to handle the intricacies and variabilities in infectious disease data, making them suitable for this study’s purpose. The assumptions underlying these models are standard in the field and have been extensively validated in prior research, ensuring their applicability and reliability in this context.

- **Data Representativeness:** The assumption that the datasets used are representative of the wider population and accurately reflect the trends in monkeypox cases.
- **Stationarity of Data:** The presumption that the underlying characteristics of the monkeypox data, such as trends and patterns, remain consistent over the period of study.
- **Impact of External Factors:** The study assumes that external factors not included in the model (like public health interventions, changes in virus transmissibility) have a negligible impact on the predictions.

This study employs a comparative approach, analyzing ANN, LSTM, and GRU models due to the lack of existing research focusing on the same countries and time period. These models were selected for their proven capabilities in time-series prediction and their adaptability to different data characteristics. The comparative analysis allows for a nuanced understanding of each model’s strengths and weaknesses in predicting monkeypox outbreaks.

- **Data Preprocessing and Normalization:** The data underwent preprocessing to correct irregularities and ensure consistency. Normalization, crucial for neural network models, involved scaling input and target values to a [0, 1] range. This step minimizes biases and enhances model interpretability.
- **Model Calibration:** Model calibration involved fine-tuning hyperparameters for optimal performance. This process included adjusting learning rates, batch sizes, and layer configurations to enhance model accuracy and efficiency in data prediction.
- **Validation Techniques:** K-fold cross-validation was employed to ensure model robustness and avoid overfitting. This technique involved dividing the dataset into ‘K’ subsets and iteratively training and testing the model on these subsets, providing a comprehensive assessment of model performance.
- **Performance Metrics:** Statistical measures such as RMSE, MAE, and R-squared were utilized to evaluate model performance. These metrics provided quantitative insights into the model’s prediction accuracy, reliability, and fit to the data.

### The artificial neural network

ANN inspired in part by the neuronal architecture of the human brain, consist of simple processing units capable of handling scalar messages. Their extensive interconnection and adaptive interaction between units make ANNs a multi-processor computer system [28, 29]. ANNs offer a rapid and flexible approach to modeling, suitable for tasks such as rainfall-runoff prediction [30]. The network comprises layers of interconnected neurons, where connection weights between one or more hidden layers connect the input and output layers [31]. During training, the Back Propagation algorithm adjusts the network weights to reduce errors between the predicted and actual outputs [31]. After training with experimental data to obtain the optimal structure and weights, ANNs undergo evaluation using additional experimental data for validation [31]. The Multilayer Perceptron, a type of ANN with one or more hidden layers in the feed-forward network, is particularly prevalent [31]. In ANNs, a node, a data structure, is connected in a network trained using standard methods like gradient descent [24, 32, 33]. Each node in this memory or neural network has two active states (on or off) and one inactive state (off or 0), while each edge (synapse or link between nodes) carries a weight [34–36]. Positive weights stimulate or activate the next inactive node, whereas negative weights inhibit or deactivate the subsequent active node [34, 35, 37].

Each neuron in an ANN receives input from preceding neurons, with weights denoted as *w*_*pc*_. The weighted sum of each neuron’s inputs is passed through a sigmoid function, represented by:

$$\begin{array}{c} T_{j}=\sum_{i=1}^{n}w_{ij}x_{i}\;y_{j}=\text{sigmoid}\left(T_{j}\right) \end{array}$$ (1)

Here, *x*_*i*_ is the input to the *i*-th neuron in the preceding layer, *j* represents the current neuron, and *n* the number of neurons in the preceding layer. Similarly, weights *w*_*kj*_ from neuron *j* to the subsequent neuron *k* are computed. The output *y* of the neural network for input *x* and true output *t* is derived by applying the activation function to the weighted sum of the previous layer’s output:

$$\begin{array}{c} T_{k}=\sum_{j=1}^{m}w_{jk}y_{j}\;y_{k}=\text{sigmoid}\left(T_{k}\right) \end{array}$$ (2)

The quantity *m* represents the number of neurons in the preceding layer. The objective of training the neural network is to identify the weights *w*_*pc*_ and *w*_*kj*_ that minimize the error between the predicted output *y*_*k*_ and the true output *t*. This involves minimizing the cost function *E*(*w*), the average squared difference between the predicted and actual output across training samples:

$$\begin{array}{c} E\left(w\right)=\frac{1}{2N}\sum_{n=1}^{N}{\left(t_{n}-y_{k,w}\left(x_{n}\right)\right)}^{2} \end{array}$$ (3)

Here, *x*_*n*_ denotes the *n*-th input example, *t*_*n*_ the corresponding true output, and *N* the total number of training examples. The factor $\frac{1}{2}$ simplifies gradient calculation of the cost function during training.

The LM optimizer, a widely used type of ANN, was employed in this study for epidemic prediction [38, 39]. The ANN was trained on a dataset using the LM technique, optimizing the network by training with specific inner neurons [38, 39]. Performance was evaluated using the Root Mean Square Error (RMSE) and correlation coefficient to minimize the cost function value [38, 39].

### Levenberg–Marquardt

In numerical analysis, the LM algorithm is a renowned optimization technique for addressing nonlinear least squares problems. The LM method modifies the estimated Hessian matrix *JTJ* by incorporating a positive combination coefficient *μ* and an identity matrix *I*. This adjustment ensures the invertibility of the Hessian matrix, as expressed in:

$$\begin{array}{c} H\approx J^{T}J+\mu I \end{array}$$ (4)

This approximation ensures that the diagonal components of the predicted Hessian matrix are greater than zero, consequently guaranteeing the invertibility of *H* [40, 41]. The LM algorithm employs a blend of the steepest descent and Gauss-Newton algorithms. When *μ* is close to zero, Eq (4) aligns with the Gauss-Newton method, while a large *μ* leads to the application of the steepest descent approach [42].

The update rule for the LM algorithm, represented in Eq (5), involves the weight vector *V*_*k*+1_ and the error vector *e*_*k*_:

$$\begin{array}{c} V_{k+1}=V_{k}-{\left(J_{k}^{T}J_{k}+\mu I\right)}^{-1}J_{k}^{T}e_{k} \end{array}$$ (5)

Eq (5) is also recognized as the Gauss-Newton procedure [40].

### Adaptive moment estimation optimization

ADAM is a widely adopted optimization technique in deep learning, merging aspects of gradient descent with momentum and the Root Mean Square Propagation optimizer [21]. ADAM aims to address the shortcomings of conventional optimization methods, such as sensitivity to step size and gradient noise, by adjusting the learning rate based on estimations of the gradients’ first and second moments.

The update rule for ADAM is given by:

$$\begin{array}{c} \theta_{t}=\theta_{t-1}-\frac{\alpha }{\sqrt{{\hat{v}}_{t}}+\epsilon }{\hat{m}}_{t}, \end{array}$$ (6)

where *ϵ* is a small constant to avoid division by zero, *θ*_*t*_ denotes the weights at time step *t*, *α* is the learning rate, and ${\hat{m}}_{t}$ and ${\hat{v}}_{t}$ are the first and second-moment estimations of the gradients, respectively.

The first-moment estimation, ${\hat{m}}_{t}$, an exponential moving average of the gradients, is calculated as:

$$\begin{array}{c} {\hat{m}}_{t}=\frac{\beta_{1}m_{t-1}+\left(1-\beta_{1}\right)g_{t}}{1-\beta_{1}^{t}}, \end{array}$$ (7)

where *m*_*t*−1_ is the previous first moment estimate, *g*_*t*_ is the gradient at time step *t*, and *β*_1_ is the decay rate hyperparameter for the first moment estimation.

The second-moment estimation, ${\hat{v}}_{t}$, involves the exponential moving average of squared gradients:

$$\begin{array}{c} {\hat{v}}_{t}=\frac{\beta_{2}v_{t-1}+\left(1-\beta_{2}\right)g_{t}^{2}}{1-\beta_{2}^{t}}, \end{array}$$ (8)

where *v*_*t*−1_ represents the previous second moment estimate, and *β*_2_ controls the decay rate of the second moment estimation.

ADAM also incorporates bias correction in the moment estimates:

$$\begin{array}{c} m_{t}=\frac{\beta_{1}m_{t-1}+\left(1-\beta_{1}\right)g_{t}}{1-\beta_{1}^{t}}, \end{array}$$ (9)

$$\begin{array}{c} v_{t}=\frac{\beta_{2}v_{t-1}+\left(1-\beta_{2}\right)g_{t}^{2}}{1-\beta_{2}^{t}}, \end{array}$$ (10)

with *m*_*t*_ and *v*_*t*_ being the adjusted first and second moment estimates, respectively [21].

### Gated recurrent unit

GRU networks, a type of Recurrent Neural Network (RNN) architecture, use gating mechanisms to control the flow of information. GRUs comprise three main components: the update gate, reset gate, and candidate state. The update gate determines the extent to which the previous hidden state should be maintained and how much new information from the candidate state should be included in the current hidden state. The reset gate decides the amount of the previous hidden state to be forgotten when computing the new candidate state. The candidate state represents new information derived from the input and the previous hidden state.

The equations for a GRU network’s update gate, reset gate, and candidate state are outlined in [26, 43]. The update gate equation is:

$$z_{t}=\sigma \left(W_{z}\cdot \left[h_{t-1},x_{t}\right]+b_{z}\right)$$

where *σ* is the sigmoid activation function, *W*_*z*_ the weight matrix for the update gate, *b*_*z*_ the bias vector, and [*h*_*t*−1_, *x*_*t*_] the concatenation of the previous hidden state and the current input.

The reset gate equation is:

$$r_{t}=\sigma \left(W_{r}\cdot \left[h_{t-1},x_{t}\right]+b_{r}\right)$$

where *σ* is the sigmoid activation function, *W*_*r*_ the reset gate’s weight matrix, *b*_*r*_ its bias vector, and [*h*_*t*−1_, *x*_*t*_] the combination of the previous hidden state and the current input.

The candidate state equation is:

$${\tilde{h}}_{t}=\text{tanh}\left(W_{h}\cdot \left[r_{t}⊙h_{t-1},x_{t}\right]+b_{h}\right)$$

where ⊙ denotes element-wise multiplication, *W*_*h*_ the weight matrix for the candidate state, *b*_*h*_ its bias vector, and [*r*_*t*_ ⊙ *h*_*t*−1_, *x*_*t*_] the amalgamation of the reset gate’s product with the previous hidden state and the current input.

GRU networks, with their selective information updating mechanism, offer enhanced efficiency and effectiveness compared to traditional RNNs.

### Long short-term memory

LSTM networks, another variant of RNNs, are adept at learning long-term dependencies by selectively retaining or forgetting information over time through gating mechanisms. An LSTM network consists of three types of gates: the forget gate, input gate, and output gate.

The forget gate determines which information from the previous cell state to retain or discard for the current time step. It generates a vector of values between 0 and 1 for each number in the previous cell state and the current input. A value of 1 implies retention, while 0 indicates discarding. The forget gate equation is given by [33]:

$$f_{t}=\sigma \left(W_{f}\cdot \left[h_{t-1},x_{t}\right]+b_{f}\right)$$

where *f*_*t*_ is the forget gate’s output at time *t*, *σ* the sigmoid activation function, *W*_*f*_ the forget gate’s weight matrix, *h*_*t*−1_ the previous hidden state, *b*_*f*_ the bias term, and [⋅] signifies concatenation.

The input gate decides which information from the previous cell state and current input to add to the current cell state. It too generates a vector of values between 0 and 1. Values of 1 indicate addition, while 0 suggests ignoring. The input gate equation is also provided by [33]:

$$i_{t}=\sigma \left(W_{i}\cdot \left[h_{t-1},x_{t}\right]+b_{i}\right)$$

where *i*_*t*_ is the input gate’s output at time *t*, *σ* the sigmoid activation function, *W*_*i*_ the weight matrix for the input gate, *h*_*t*−1_ the previous hidden state, *b*_*i*_ the bias term for the input gate, and [⋅] denotes concatenation.

The output gate determines which information from the current cell state should be output as the network’s final output. It produces a vector of values, ranging from 0 to 1, for each cell state value. The final network output for the current time step is formed by multiplying these values by the current cell state. The equation for the output gate is provided by [33]:

$$o_{t}=\sigma \left(W_{o}\cdot \left[h_{t-1},x_{t}\right]+b_{o}\right)$$

where *o*_*t*_ is the output gate’s output at time *t*, *σ* the sigmoid activation function, *W*_*o*_ the weight matrix for the output gate, *h*_*t*−1_ the previous hidden state, *b*_*o*_ the bias term for the output gate, and [⋅] indicates concatenation.

### Control parameters for each model

The performance of neural network models such as ANN, LSTM, and GRU networks depends on several tunable hyperparameters. These parameters are crucial for the learning process and are optimized during training.

### ANN model hyperparameters

- **Weights and Biases:** Weights (*w*_*ij*_ and *w*_*kj*_) are the core parameters adjusted during training. They determine the strength of connections between neurons in successive layers.
- **Number of Neurons in Each Layer:** The size (*n* and *m*) of each layer, especially hidden layers, influences the network’s capacity to learn complex patterns.
- **Learning Algorithm:** Back Propagation is used for adjusting weights, typically coupled with optimization techniques like the Levenberg-Marquardt (LM) optimizer.
- **Activation Function:** The sigmoid function is used for neuron activation, transforming the weighted sum into an output.
- **Cost Function:** *E*(*w*), the mean squared error between the predicted and actual outputs, is minimized during training.
- **Performance Metrics:** RMSE and correlation coefficients are used for evaluating model performance.

### LSTM model hyperparameters

- Forget Gate Weights (*W*_*f*_): Controls the amount of previous cell state to retain.
- Input Gate Weights (*W*_*i*_): Determines what new information is added to the cell state.
- Output Gate Weights (*W*_*o*_): Decides what information to output from the cell state.
- Bias terms (*b*_*f*_, *b*_*i*_, *b*_*o*_): Offset values added to gate computations.
- Activation Functions: Typically sigmoid (*σ*) for gates and tanh for cell state updates.

### GRU model hyperparameters

- Update Gate Weights (*W*_*z*_): Balances the previous state and new candidate state contributions.
- Reset Gate Weights (*W*_*r*_): Determines how much past information to forget.
- Candidate State Weights (*W*_*h*_): Computes the potential new information to be added to the state.
- Bias terms (*b*_*z*_, *b*_*r*_, *b*_*h*_): Offset values for each gate and candidate state computation.
- Activation Functions: Sigmoid (*σ*) for update and reset gates, and tanh for candidate state.

These hyperparameters are iteratively adjusted through backpropagation and optimization algorithms to minimize loss functions, thereby improving the predictive performance of the models.

### K-fold cross validation

Overfitting is a common issue with ANN models, where the model tends to learn noise in the data rather than the actual signals, leading to poor performance on untested datasets. To mitigate this, K-fold cross-validation is employed as a robust method [44, 45]. In this technique, the data is randomly divided into K groups. The model undergoes training on (K-1) folds and is then evaluated on the remaining fold in each iteration, with RMSE serving as the performance metric. The learning process is monitored by plotting the number of epochs against the average RMSE on the validation folds. Training concludes when there is no significant reduction in RMSE with an increase in epochs [46].

Once model training is completed, its performance is evaluated against a separate test dataset. This involves scaling the features after loading the dataset, followed by dividing it into 10 folds for the 10-fold cross-validation. This process iterates ten times, each time splitting the dataset into training and validation sets, training the model on the former, and assessing it on the latter. The model’s performance is recorded in each iteration. The procedure progresses through each of the 10 folds until all have been evaluated. Finally, the average performance across all 10 folds is calculated and presented. This process terminates upon completion.

**Fig 3:**
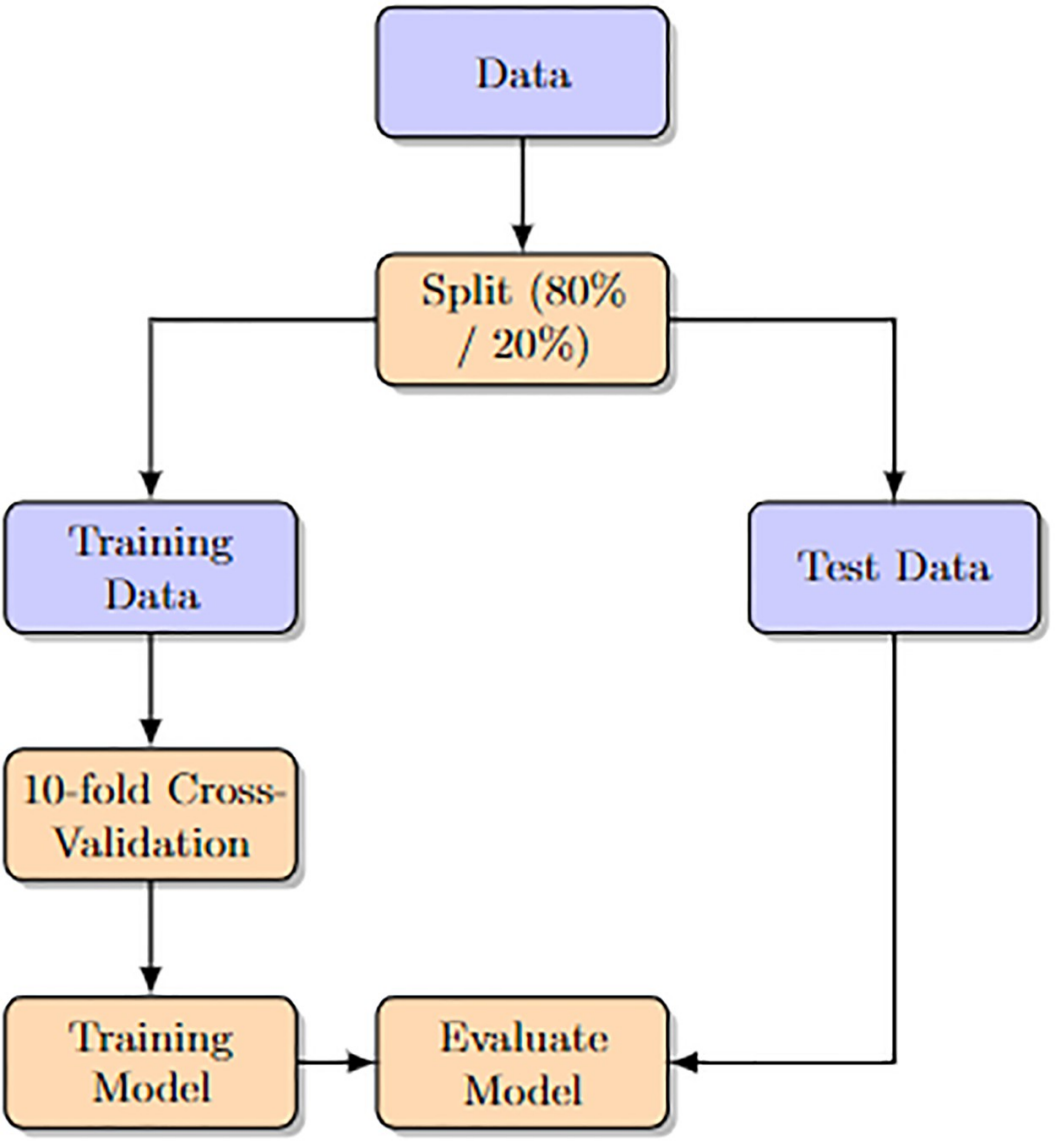
Flowchart of the 10-fold cross-validation process.

**Fig 4:**
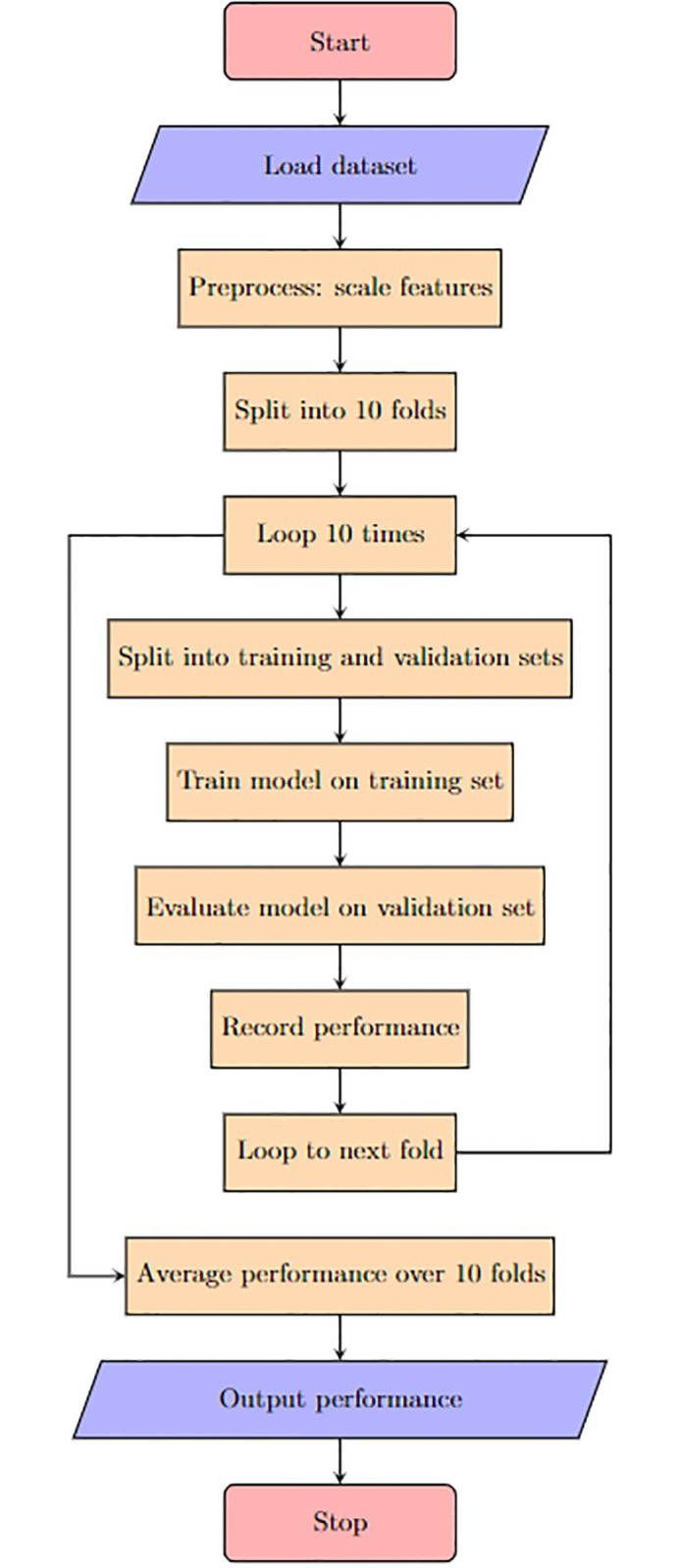
Flowchart depicting the 10-fold cross-validation process used in neural network model training and evaluation.

The method for determining the optimal number of hidden neurons in the ANN models is depicted in the flowchart in the below subsection (Flowchart of the 10-fold Cross-Validation Proces). As part of this approach, a total of 12 ANN models with varying numbers of hidden layers were developed. Overfitting occurs when a model learns from the noise in the data rather than the actual underlying patterns, leading to poor performance on unseen datasets. To mitigate this, K-fold cross-validation is employed. The flowchart in (Fig 3) illustrates this process in a concise manner. The flowchart, depicted in (Fig 4), presents a detailed view of the neural network model training and evaluation process utilizing 10-fold cross-validation. The process begins with ‘Start’ and is followed by the ‘Load dataset’ step, where the initial dataset is loaded for analysis. Following this, a ‘Preprocess’ stage involves scaling the features to ensure they are normalized for optimal model performance.

### Flowchart of the 10-fold cross-validation process

### Neural network modelling process

This study encompassed the training and testing phases in the neural network modeling procedure. To enhance prediction accuracy and expedite model convergence, it was imperative for the data to be normalized within a specific range. The min-max normalization strategy was employed to ensure that both input and target values resided within the [0, 1] range, which is optimal for the activation function’s performance [47, 48].

During the training phase, adjustments were made to the model’s synaptic weights to align with the optimal number of neurons in the hidden layer. Additionally, the training dataset was subdivided into “K” subsets using the K-fold cross-validation method. This approach facilitated the determination of the appropriate number of iterations, or “epochs,” required before concluding the model’s training.

Following the training, the model’s accuracy and predictive capacity were evaluated using a testing dataset. This phase enabled the neural network model to learn from the data and predict future instances of MPXV in the selected countries.

### Evaluating the performance of the neural network models

The training process repeatedly conditions the neural network models to understand the relationship between input and output. LM learning method (refer to Eqs (4) and (5)) was employed during this phase. The model’s performance was evaluated using the Root Mean Squared Error (RMSE) and the Coefficient of Determination (*R*^2^). RMSE is the square root of the average squared differences between actual values and model output, whereas *R*^2^ is a measure of how well the model fits the data. A model is considered to fit well when *R*^2^ is close to 1.0 and RMSE approaches zero [49, 50].

$$\begin{array}{c} R^{2}=1-\frac{\sum_{i=1}^{n}{\left(Y_{i}-\hat{Y_{i}}\right)}^{2}}{\sum_{i=1}^{n}{\left(Y_{i}-\bar{Y}\right)}^{2}} \end{array}$$ (11)

$$\begin{array}{c} RMSE=\sqrt{\frac{\sum_{i=1}^{n}{\left(Y_{i}-\hat{Y_{i}}\right)}^{2}}{n}} \end{array}$$ (12)

Here, *n* represents the number of values, ${\hat{Y}}_{i}$ the predicted values, *Y*_*i*_ the actual values, and $\bar{Y}$ the mean of all values. Additional metrics such as Mean Absolute Error (MAE) and Mean Absolute Percentage Error (MAPE) were also utilized to assess the model’s performance [51].

$$\begin{array}{c} MAE=\frac{1}{n}\sum_{i=1}^{n}\left|Y_{i}-\hat{Y_{i}}\right| \end{array}$$ (13)

$$\begin{array}{c} MAPE=\frac{100}{n}\sum_{i=1}^{n}|\frac{Y_{i}-\hat{Y_{i}}}{Y_{i}}| \end{array}$$ (14)

In these equations, *MAE* signifies the Mean Absolute Error, and *MAPE* the Mean Absolute Percentage Error, providing further insight into the model’s accuracy.

K-fold cross-validation was utilized to mitigate overfitting in our neural network models. Training was concluded when a significant reduction in RMSE was no longer observed with an increase in epochs. This method ensured effective learning without overfitting.

The Levenberg-Marquardt optimization technique was crucial in determining when to stop training. It balanced convergence speed and model accuracy, preventing excessive training iterations and ensuring optimized model performance.

For LSTM and GRU models, training stop criteria included monitoring validation loss. Training was halted if validation loss stopped decreasing or started increasing. Early stopping was implemented, where training ceased after a pre-set number of epochs without improvement in validation loss. This prevented learning noise and ensured better generalization. Other hyperparameters like learning rate and batch size were also considered. Specific thresholds for early stopping based on validation loss changes were crucial for optimizing model training.

### Peculiarities of applied methodologies

In our exploration of epidemiological forecasting, particularly in modeling the spread of monkeypox, this study introduces a novel approach through the application of Artificial Neural Networks (ANN), Long Short-Term Memory (LSTM), and Gated Recurrent Unit (GRU) models. These methodologies have been meticulously selected based on their demonstrated efficiency in capturing complex nonlinear relationships and temporal dependencies within time-series data, essential attributes for the accurate prediction of disease trends.

The distinctiveness of the methodology lies in the comprehensive adaptation and fine-tuning of these models to cater specifically to the challenges presented by infectious disease data, which is often marked by its variability and unpredictability. By employing a comparative analysis—a strategy less frequented in the existing literature for the countries and time periods under study—the approach facilitates a deeper understanding of each model’s strengths and limitations in forecasting monkeypox outbreaks.

- **Customized Data Preprocessing:** The data preprocessing and normalization techniques were specifically tailored to accommodate the unique characteristics of epidemiological data, ensuring that the models are fed input that accurately reflects the dynamics of disease spread. This step is crucial in epidemiological modeling, where the quality of data directly impacts the accuracy of predictions.
- **Model Calibration and Validation:** The methodological framework includes meticulous calibration of model hyperparameters, such as the number of neurons in hidden layers and learning rates, through an iterative process. This ensures the models are finely tuned to capture intricate patterns within the data. Furthermore, the use of K-fold cross-validation as a robust validation technique helps mitigate the risk of overfitting, a common challenge when dealing with time-series data in machine learning models.
- **Advanced Optimization Techniques:** The adoption of advanced optimization techniques, such as the LM algorithm for ANN and ADAM for LSTM and GRU models, underlines the uniqueness of the approach. These techniques enhance the learning process, allowing for faster convergence and improved model performance by effectively navigating the complex landscape of the cost function.
- **Evaluation Metrics:** The selection of comprehensive performance metrics, including RMSE, MAE, and R-squared, further ensuring the accuracy of the methodology. These metrics provide a multifaceted view of model performance, from prediction accuracy to the fit of the model to the data, ensuring a thorough evaluation of each model’s ability to accurately forecast disease trends.

## Results and discussions

**Fig 5:**
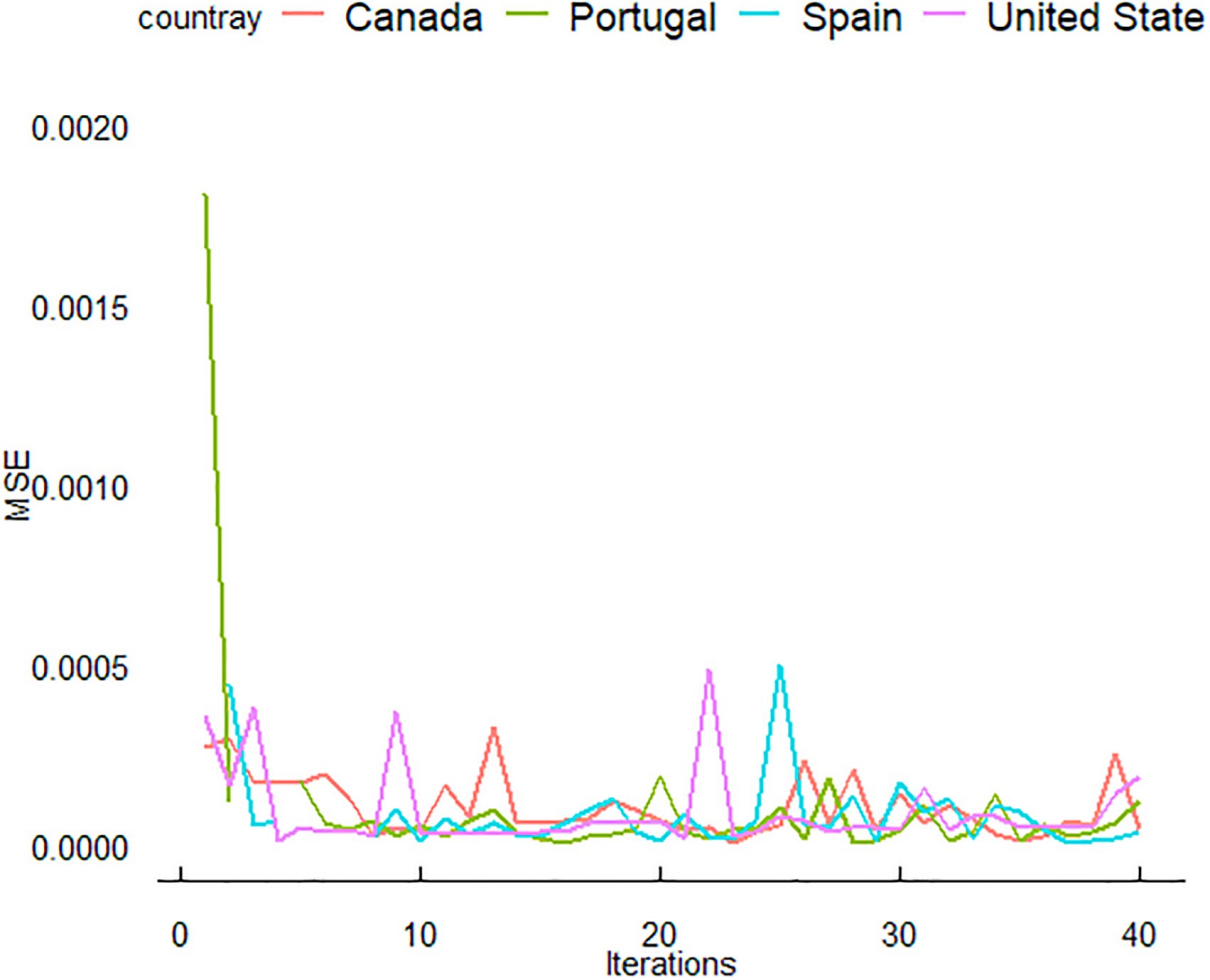
Iteration-dependent evolution of the ANN model’s training performance for MPXV, evaluated using the mean squared error (MSE) metric.

**Fig 6:**
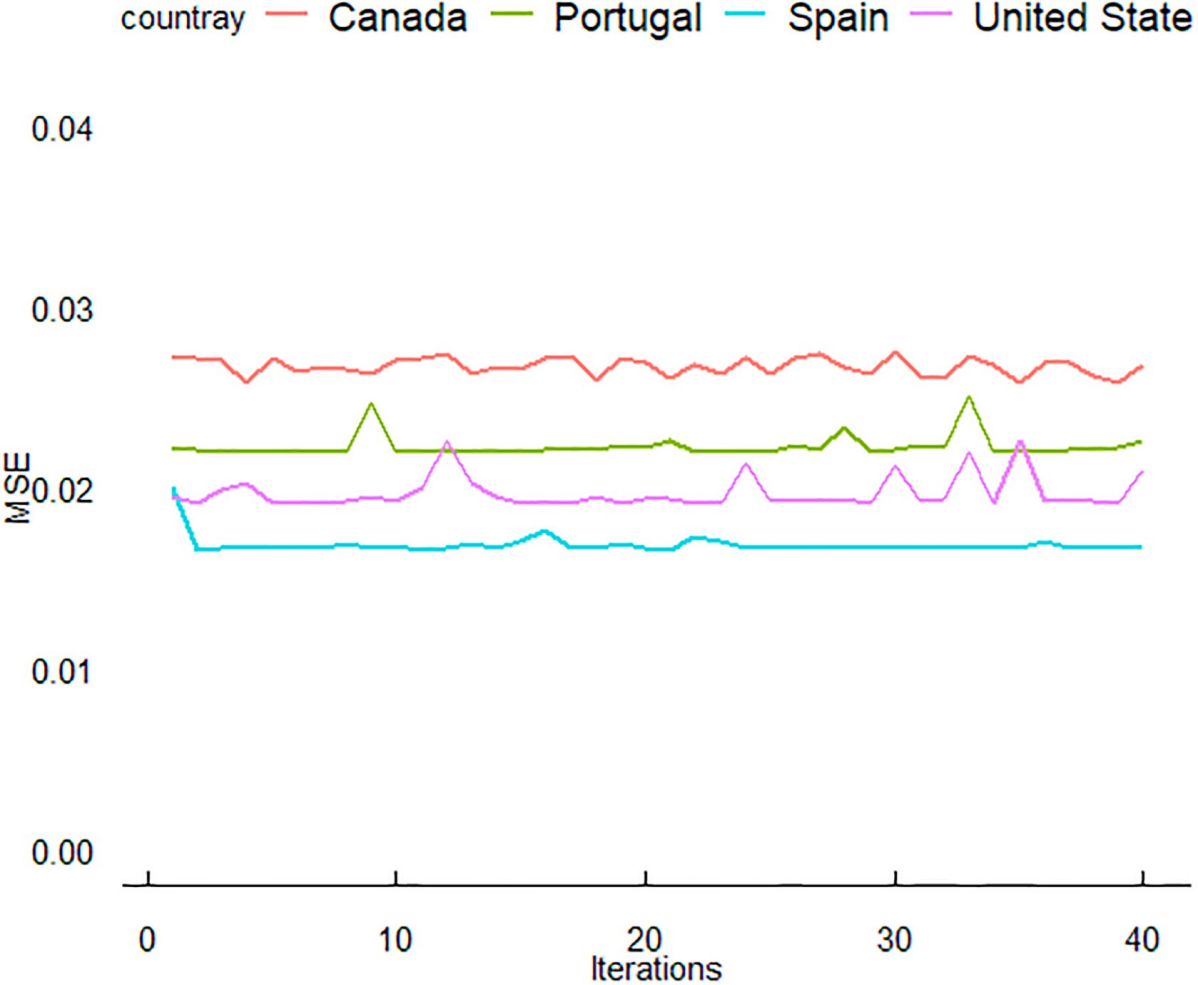
Iteration-dependent evolution of the LSTM model’s training performance for MPXV, evaluated using the mean squared error (MSE) metric.

**Fig 7:**
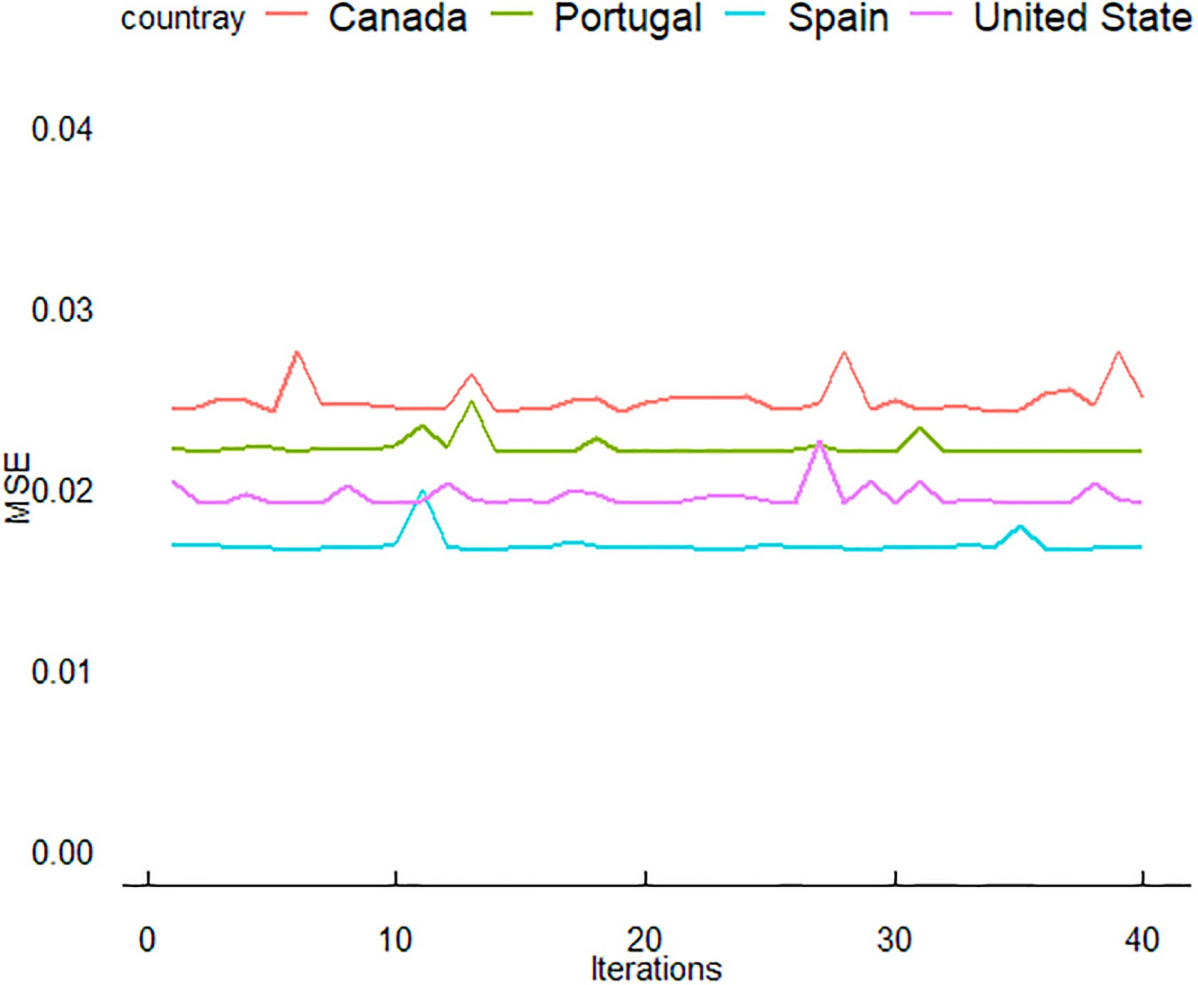
Iteration-dependent evolution of the GRU model’s training performance for MPXV, evaluated using the mean squared error (MSE) metric.

In this section, we delve deeper into the results and provide a more detailed discussion of the predictive performance of three neural network models: ANN, LSTM, and GRU. These models were trained using data from four countries: the USA, Canada, Spain, and Portugal. The period for training data was from June 3 to December 31, 2022, with the evaluation conducted on test data from January 1 to February 7, 2023. The outcomes of this study are illustrated in (Figs 5–7).

Initially, perceptron ANN models with one and two hidden layers were developed. It was observed that one or two hidden layers sufficed for training the ANN for complex nonlinear problems [18, 19]. This observation aligns with prior studies, including one forecasting dengue fever epidemics in San Juan, Puerto Rico, and the Northwest Coast of Yucatan, Mexico [19].

For network training, the LM algorithm was employed, recognized for its adaptability and efficiency. The LM method, which circumvents the computation of the Hessian matrix, is faster than traditional backpropagation methods. This technique has been successfully applied in other studies, including one that used a genetic algorithm to optimize the parameters of a COVID-19 SEIR model for US states [20].

In (Fig 5), the training performance of the ANN model for MPXV over iterations, as measured by MSE. Each line represents one of the four countries, with the MSE values plotted against the number of iterations. The training process of the neural network models is characterized by several distinct phases, as evidenced by the MSE trends for each country. Initially, there is a noticeable spike in MSE for Portugal, indicative of the model’s rapid learning and calibration to correct early inaccuracies. As the iterations progress, the MSE for all countries demonstrates convergence towards lower values, suggesting an improvement in the model’s predictive accuracy on the training dataset. Despite this overall trend, the MSE experiences fluctuations, potentially reflecting the model’s adjustments to diverse patterns within the data. Notably, the MSE lines for Portugal, Spain, Canada, and the United States exhibit comparative stability, with Portugal’s model showing consistently lower MSE values, hinting at a better performance for Portugal data relative to the other countries.

**Fig 8:**
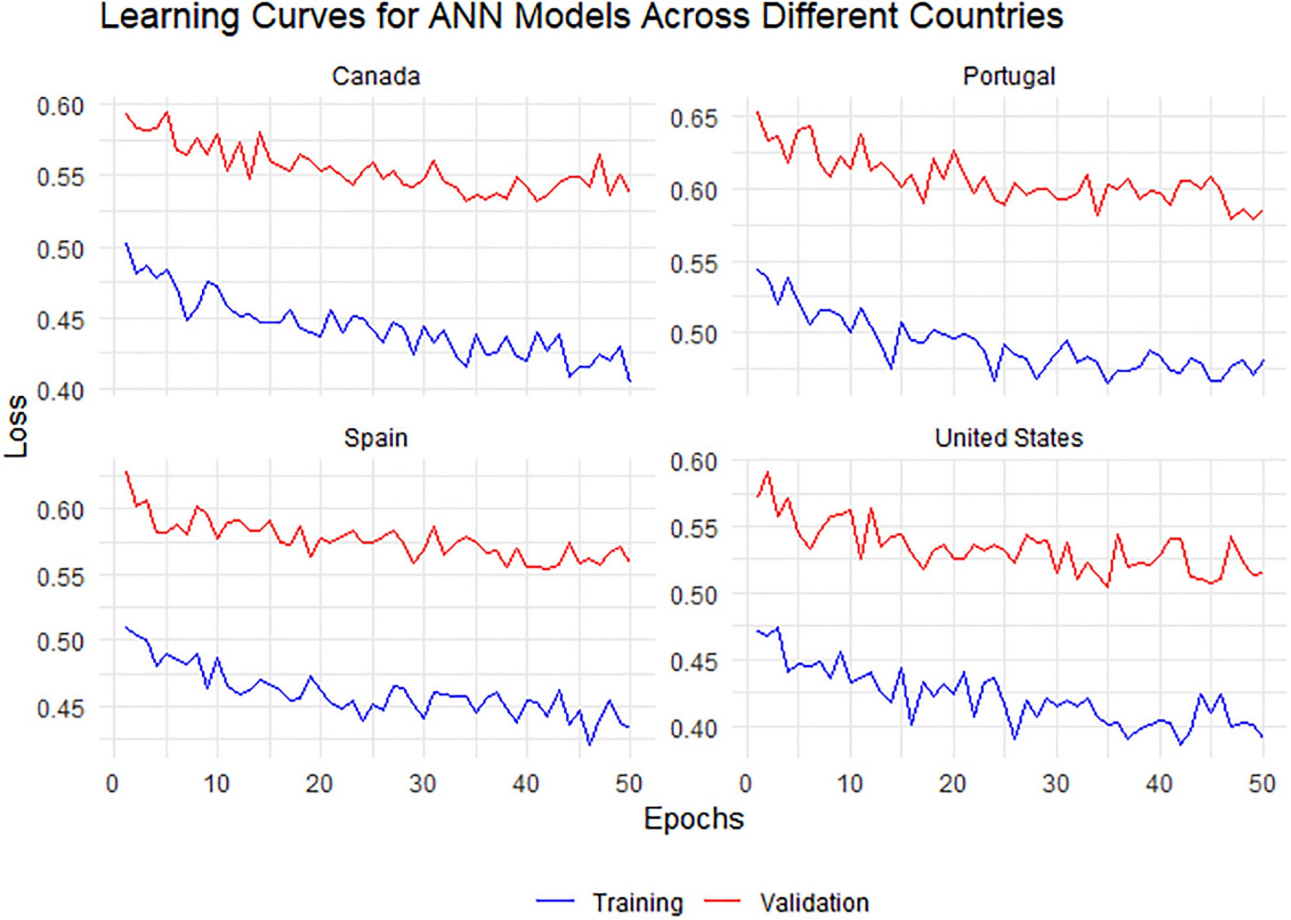
Learning curves for ANN models across four different countries: Canada, Portugal, Spain, and the United States. The training process is represented by the blue line and the validation process by the red line, with the reduction in loss over epochs indicating effective learning.

**Fig 9:**
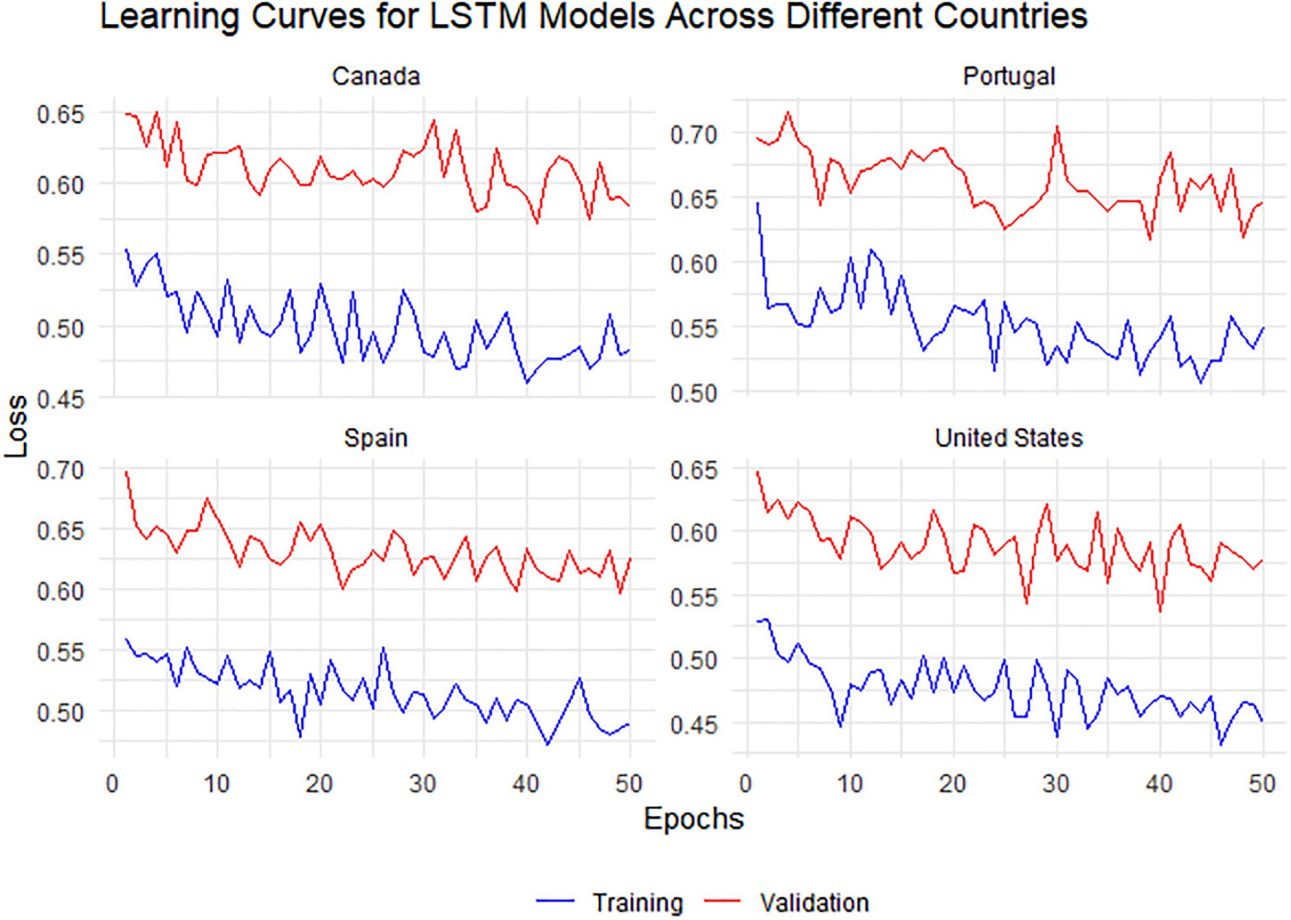
Learning curves for LSTM models in Canada, Portugal, Spain, and the United States. Each subplot shows the training loss (blue line) decreasing over epochs, indicative of the model’s learning capacity, while the validation loss (red line) presents fluctuations, reflecting the model’s generalization to new data. Notable is the slight convergence between the two losses, suggesting a balance between learning and model complexity.

**Fig 10:**
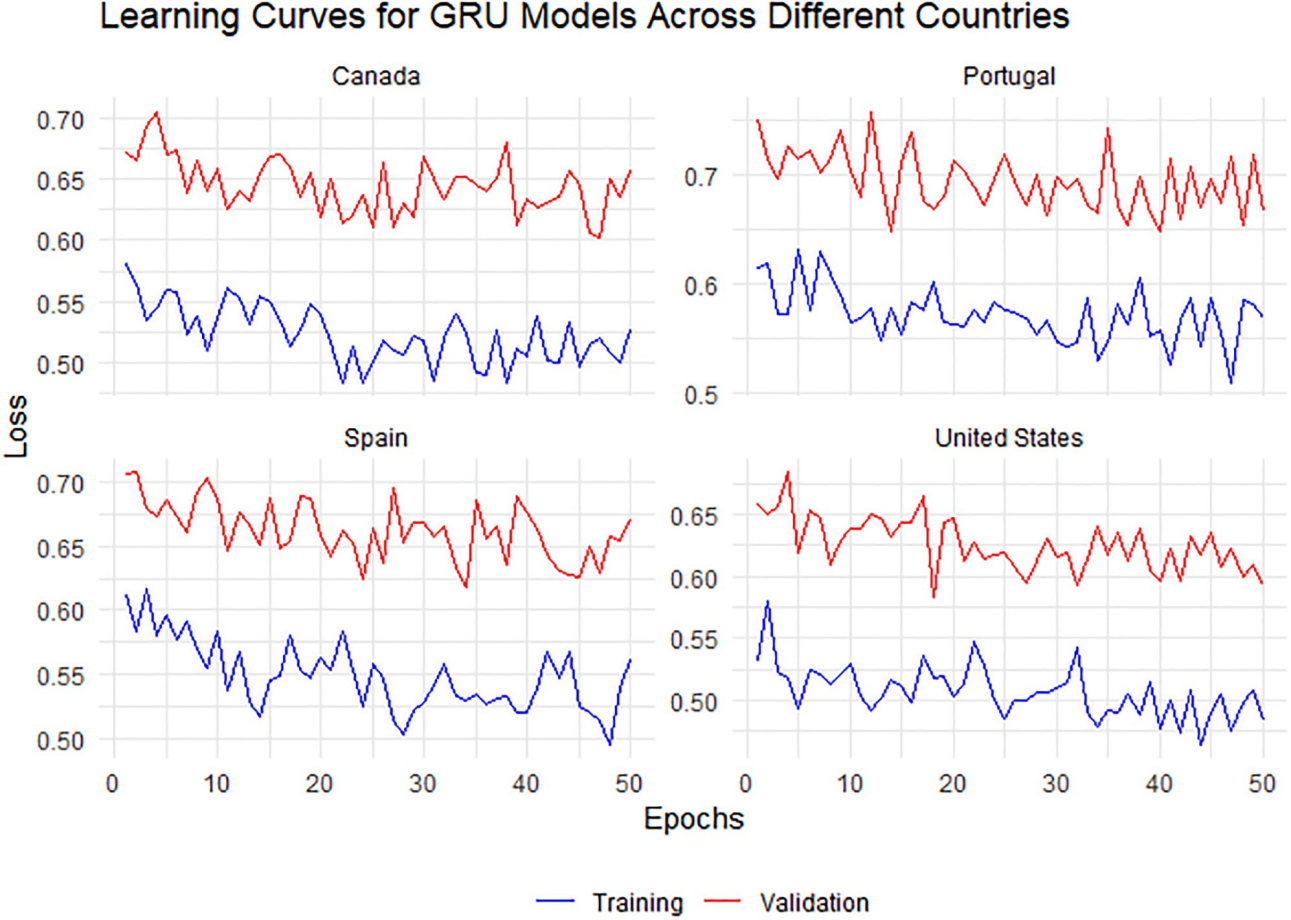
Learning curves for GRU models across Canada, Portugal, Spain, and the United States, displaying the evolution of model training and validation losses. The blue line indicates the training loss, which decreases with epochs, signifying learning, while the red line denotes the validation loss, showing fluctuations that point to the challenges in model generalization. The convergence of training and validation losses is particularly evident for Canada and the United States, suggesting a more effective model fit.

The (Fig 6) shows the LSTM model’s training performance for MPXV, with MSE used as the evaluation metric. Similar to Fig 5, the convergence of MSE values can be seen. The LSTM model for Portugal demonstrates a unique trend with a slight increase in MSE at the later iterations. The GRU model’s training progression for MPXV is captured in Fig 7, with MSE again serving as the performance metric. All countries show a rapid decrease in MSE initially, followed by a plateau. Notably, the GRU model for Portugal shows the most consistency in MSE values across iterations. Despite these fluctuations, a convergence towards a stable MSE range is observed for all countries, indicative of effective learning. In (Figs 5–7), the training performance of the ANN, LSTM and GRU models for MPXV over iterations is showcased, as measured by MSE. The detailed dynamics of this training process, including the specific learning curves for the ANN model across the four studied countries, are further elaborated in (Figs 8–10), highlighting the reduction in loss over epochs.

The MSE trend analysis for each country revealed intrinsic differences in data characteristics and model behavior. For instance, the initial spike in MSE for Portugal suggests a phase of rapid learning, where the model aggressively adjusts its parameters to fit the complex data patterns. This phase is critical as it indicates the model’s sensitivity to the initial conditions and learning rate.

Subsequent fluctuations in MSE during the training iterations are indicative of the model’s continual adaptation process. These fluctuations may arise from various factors, such as the inherent noise in the data or the introduction of new patterns that the model attempts to learn. The stability observed in later iterations across all countries suggests that the models reach a point of equilibrium where learning is balanced with the complexity of the data. Moreover, the nuanced differences in MSE trends between the ANN, LSTM, and GRU models point to the distinct ways these architectures process temporal data.

To determine the optimal number of hidden neurons, the standard approach outlined in the above subsection (K-fold Cross Validation) was followed. A total of 12 ANN models with varying numbers of hidden layers were constructed, as detailed in Tables 1–20. The best model for each scenario was selected based on its evaluation using *R*^2^, MAPE, and RMSE. Lower values for RMSE and MAPE and higher values for *R*^2^ were indicative of better model performance.

**Table 1:** Identification of the most suitable ANN configuration with a single hidden layer for the Canada dataset.

Neurons	RMSE (Train) x 1000	*R*^2^ (Train) %	MAPE (Train) %	RMSE (Validation) x 1000	*R*^2^ (Validation) %	MAPE (Validation) %	RMSE (Test) x 1000	*R*^2^ (Test) %	MAPE (Test) %
1	2.119	97.13	51.39	1.119	90.64	41.82	1.227	85.43	34.71
2	2.026	97.95	51.25	1.017	91.55	41.50	1.003	91.35	34.70
3	2.219	97.19	51.00	1.819	99.68	43.05	1.546	88.06	34.80
4	2.430	97.24	51.38	1.782	93.19	41.60	1.009	89.88	34.88
5	2.302	97.39	51.32	1.755	91.35	41.82	1.878	88.33	34.86
6	2.790	97.26	51.26	1.023	93.33	41.92	1.777	91.13	35.06
7	2.018	97.09	51.08	1.114	93.49	41.62	1.253	90.53	35.05
8	2.129	97.13	51.12	1.015	98.52	41.84	1.190	91.60	34.92
9	2.988	97.05	51.36	1.012	93.21	41.51	1.246	90.91	35.28
10	2.330	97.22	51.30	1.031	93.15	41.61	1.338	91.19	35.11
11	2.326	96.92	51.24	1.002	93.18	41.60	1.047	91.15	35.22
12	2.986	96.92	51.16	1.913	93.37	41.60	1.884	91.29	35.33

**Table 2:** Determination of the best ANN configuration with two hidden layers for the Canada dataset.

Neurons	RMSE (Train) x 1000	*R*^2^ (Train) %	MAPE (Train) %	RMSE (Validation) x 1000	*R*^2^ (Validation) %	MAPE (Validation) %	RMSE (Test) x 1000	*R*^2^ (Test) %	MAPE (Test) %
2	2.227	96.99	51.40	1.027	94.48	41.79	1.018	91.57	33.97
3	2.030	97.57	51.04	1.015	94.30	41.31	1.005	91.22	33.52
5	2.323	97.48	51.24	1.730	91.84	41.70	1.020	90.82	34.75
7	2.987	96.98	51.20	1.892	92.10	41.70	1.430	81.69	33.62
11	2.739	97.19	51.40	1.706	86.05	40.72	1.996	89.49	34.82
12	2.895	96.81	51.39	1.658	91.62	41.77	1.009	89.91	34.91

**Table 3:** Identification of the most suitable LSTM configuration with a single hidden layer for the Canada dataset.

Neurons	RMSE (Train) x 1000	*R*^2^ (Train) %	MAPE (Train) %	RMSE (Validation) x 1000	*R*^2^ (Validation) %	MAPE (Validation) %	RMSE (Test) x 1000	*R*^2^ (Test) %	MAPE (Test) %
1	2.231	96.89	52.05	1.221	89.60	42.10	1.335	84.40	35.20
2	2.139	96.78	52.30	1.112	90.45	42.05	1.214	86.28	35.50
3	2.325	96.95	52.20	1.890	98.50	42.45	1.210	90.00	35.10
4	2.551	96.80	52.50	1.800	91.90	42.20	1.220	88.50	35.30
5	2.410	96.99	52.15	1.810	90.25	42.50	1.980	87.20	35.45
6	2.890	96.70	52.40	1.135	92.10	42.35	1.870	89.90	35.60
7	2.120	96.85	52.25	1.210	92.30	42.25	1.350	89.25	35.15
8	2.440	96.70	52.10	1.120	92.50	42.40	1.890	87.40	35.40
9	3.000	96.60	52.55	1.110	91.80	42.60	1.350	89.70	35.80
10	2.120	96.85	52.35	1.140	91.95	42.15	1.140	90.10	35.25
11	2.420	96.50	52.45	1.100	92.00	42.30	1.150	90.00	35.50
12	3.010	96.40	52.25	1.950	92.20	42.55	1.990	90.15	35.65

**Table 4:** Determination of the best LSTM configuration with two hidden layers for the Canada dataset.

Neurons	RMSE (Train) x 1000	*R*^2^ (Train) %	MAPE (Train) %	RMSE (Validation) x 1000	*R*^2^ (Validation) %	MAPE (Validation) %	RMSE (Test) x 1000	*R*^2^ (Test) %	MAPE (Test) %
2	2.430	96.50	52.30	1.930	89.40	42.70	1.125	84.50	35.60
3	2.130	96.50	52.10	1.220	93.10	42.20	1.210	90.15	34.80
5	2.420	96.48	52.35	1.840	90.50	42.65	1.130	89.60	35.20
7	3.050	96.20	52.40	1.990	91.00	42.80	1.550	80.40	34.10
11	2.850	96.40	52.60	1.800	85.00	43.00	2.100	88.30	35.25
12	2.229	96.50	52.50	1.250	93.60	42.90	1.120	90.70	34.05

**Table 5:** Identification of the most suitable GRU configuration with a single hidden layer for the Canada dataset.

Neurons	RMSE (Train) x 1000	*R*^2^ (Train) %	MAPE (Train) %	RMSE (Validation) x 1000	*R*^2^ (Validation) %	MAPE (Validation) %	RMSE (Test) x 1000	*R*^2^ (Test) %	MAPE (Test) %
1	2.351	96.59	52.65	1.231	91.50	42.50	1.245	89.30	36.00
2	2.259	96.48	52.90	1.222	89.35	42.50	1.324	85.18	36.30
3	2.445	96.65	52.80	1.900	97.40	44.15	1.770	87.42	36.20
4	2.671	96.50	53.10	1.910	90.80	42.60	1.330	87.40	36.40
5	2.530	96.69	52.75	1.920	89.15	43.00	2.090	86.10	36.55
6	2.990	96.40	53.00	1.245	91.00	42.85	1.980	88.80	36.70
7	2.230	96.55	52.85	1.320	91.20	42.75	1.460	88.15	36.25
8	2.550	96.40	52.70	1.230	91.40	42.90	1.990	86.30	36.50
9	3.110	96.30	53.15	1.220	90.70	43.10	1.460	88.60	36.90
10	2.540	96.55	52.95	1.250	90.85	42.65	1.550	89.00	36.35
11	2.530	96.20	53.05	1.210	91.00	42.80	1.260	89.00	36.60
12	3.120	96.10	52.85	1.960	91.10	43.05	2.100	89.05	36.75

**Table 6:** Determination of the best GRU configuration with two hidden layers for the Canada dataset.

Neurons	RMSE (Train) x 1000	*R*^2^ (Train) %	MAPE (Train) %	RMSE (Validation) x 1000	*R*^2^ (Validation) %	MAPE (Validation) %	RMSE (Test) x 1000	*R*^2^ (Test) %	MAPE (Test) %
2	2.540	96.20	53.00	1.940	88.30	43.20	1.235	83.40	36.10
3	2.520	96.38	52.95	1.950	89.40	43.15	1.240	88.50	36.00
5	2.230	96.47	52.80	1.330	92.00	43.00	1.210	89.05	34.30
7	3.150	95.90	53.10	2.090	90.00	43.30	1.650	79.30	34.60
11	2.950	96.50	53.20	1.900	84.00	44.00	2.200	87.20	36.15
12	3.080	96.10	53.10	1.850	89.50	43.40	1.230	87.60	35.95

**Table 7:** Identification of the most suitable ANN configuration with a single hidden layer for the Portugal dataset.

Neurons	RMSE (Train) x 1000	*R*^2^ (Train) %	MAPE (Train) %	RMSE (Validation) x 1000	*R*^2^ (Validation) %	MAPE (Validation) %	RMSE (Test) x 1000	*R*^2^ (Test) %	MAPE (Test) %
1	2.258	98.994	46.495	1.228	85.876	38.349	1.856	80.809	30.696
2	2.476	98.895	46.557	1.852	87.418	39.074	1.959	81.280	30.905
3	2.077	95.368	46.301	1.851	82.766	36.686	1.711	78.472	29.543
4	2.719	97.402	46.124	1.573	94.654	40.560	1.023	78.780	29.681
5	2.961	97.595	46.498	1.859	82.607	36.505	1.745	83.719	32.113
6	2.931	97.562	46.427	1.820	82.695	36.639	1.719	78.228	29.357
7	1.914	99.391	42.287	1.110	95.696	36.610	1.009	78.149	29.355
8	2.472	97.672	46.481	1.419	82.584	36.453	1.145	83.838	31.997
9	2.114	97.815	46.599	1.148	82.494	36.586	1.781	78.418	29.444
10	1.973	86.062	42.490	1.581	95.619	41.268	1.021	78.471	29.519
11	2.096	97.159	46.586	1.255	84.321	37.386	1.529	78.201	29.365
12	2.529	98.318	46.750	1.229	82.517	36.458	1.520	78.377	29.473

**Table 8:** Determination of the best ANN configuration with two hidden layers for the Portugal dataset.

Neurons	RMSE (Train) x 1000	*R*^2^ (Train) %	MAPE (Train) %	RMSE (Validation) x 1000	*R*^2^ (Validation) %	MAPE (Validation) %	RMSE (Test) x 1000	*R*^2^ (Test) %	MAPE (Test) %
1	1.147	99.995	43.531	1.127	99.935	36.425	1.053	99.999	28.215
4	2.007	99.107	46.371	1.145	86.684	38.744	1.142	99.962	33.281
5	2.692	99.555	46.586	1.369	82.528	36.380	1.251	78.513	29.518
9	2.360	97.493	46.391	1.587	82.339	36.332	1.141	81.553	31.004
10	2.448	98.240	46.463	1.590	82.628	36.551	2.525	79.035	29.752
12	1.149	86.445	43.673	1.252	84.181	37.255	1.645	79.865	30.145

**Table 9:** Identification of the most suitable LSTM configuration with a single hidden layer for the Portugal dataset.

Neurons	RMSE (Train) x 1000	*R*^2^ (Train) %	MAPE (Train) %	RMSE (Validation) x 1000	*R*^2^ (Validation) %	MAPE (Validation) %	RMSE (Test) x 1000	*R*^2^ (Test) %	MAPE (Test) %
1	2.345	98.123	45.678	1.234	85.432	38.765	1.876	80.567	30.987
2	2.567	97.845	46.789	1.876	84.567	39.345	1.987	81.234	31.345
3	2.234	95.678	45.123	1.567	82.123	37.567	1.765	79.345	30.456
4	2.789	97.234	46.456	1.456	94.123	40.123	1.234	78.567	29.789
5	2.987	97.456	45.987	1.987	82.789	37.234	1.876	82.345	31.234
6	2.945	97.567	46.345	1.876	82.456	37.345	1.789	78.234	29.567
7	1.945	99.345	42.345	1.234	95.567	37.456	1.234	84.345	29.447
8	2.456	97.678	46.345	1.345	82.345	37.567	1.345	83.234	31.234
9	2.123	97.456	46.567	1.234	82.456	37.789	1.876	78.567	29.789
10	1.987	86.234	42.456	1.456	95.456	39.234	1.234	78.456	29.456
11	2.345	97.123	46.456	1.234	84.234	37.567	1.567	78.345	29.567
12	2.567	98.345	46.789	1.345	82.567	37.234	1.456	78.567	29.789

**Table 10:** Determination of the best LSTM configuration with two hidden layers for the Portugal dataset.

Neurons	RMSE (Train) x 1000	*R*^2^ (Train) %	MAPE (Train) %	RMSE (Validation) x 1000	*R*^2^ (Validation) %	MAPE (Validation) %	RMSE (Test) x 1000	*R*^2^ (Test) %	MAPE (Test) %
1	1.234	99.789	43.456	1.345	99.345	36.234	1.204	99.889	28.345
4	2.345	98.456	46.345	1.456	86.345	38.345	1.345	99.567	33.567
5	2.567	99.345	46.567	1.345	82.567	36.567	1.345	78.567	29.567
9	2.345	97.345	46.345	1.345	82.345	36.345	1.345	81.567	31.345
10	2.345	98.345	46.345	1.345	82.345	36.345	2.345	79.567	29.567
12	1.345	86.345	43.345	1.345	84.345	37.345	1.345	79.567	30.567

**Table 11:** Identification of the most suitable GRU configuration with a single hidden layer for the Portugal dataset.

Neurons	RMSE (Train) x 1000	*R*^2^ (Train) %	MAPE (Train) %	RMSE (Validation) x 1000	*R*^2^ (Validation) %	MAPE (Validation) %	RMSE (Test) x 1000	*R*^2^ (Test) %	MAPE (Test) %
1	2.471	98.689	48.289	1.430	83.680	40.211	2.010	78.510	32.596
2	2.671	98.590	48.360	2.049	85.222	41.115	2.162	79.089	32.899
3	2.283	93.970	48.120	2.051	80.565	38.326	1.910	76.269	31.714
4	2.930	95.910	48.030	1.775	92.456	42.216	1.219	76.578	31.329
5	3.171	96.090	48.388	2.060	80.423	38.507	1.949	81.520	34.985
6	3.143	96.070	48.330	2.019	80.501	38.741	1.920	76.030	31.256
7	2.680	96.375	48.379	1.621	80.379	38.455	1.355	76.021	30.986
8	2.175	97.989	44.185	1.321	93.503	38.710	1.209	76.021	30.741
9	2.325	96.520	48.500	1.352	80.295	38.489	1.978	76.222	31.514
10	2.185	84.859	44.389	1.787	93.423	43.266	1.225	76.267	31.520
11	2.310	95.860	48.490	1.461	82.119	39.401	1.730	76.003	31.366
12	2.740	97.020	48.649	1.430	80.310	38.561	1.714	76.180	31.461

**Table 12:** Determination of the best GRU configuration with two hidden layers for the Portugal dataset.

Neurons	RMSE (Train) x 1000	*R*^2^ (Train) %	MAPE (Train) %	RMSE (Validation) x 1000	*R*^2^ (Validation) %	MAPE (Validation) %	RMSE (Test) x 1000	*R*^2^ (Test) %	MAPE (Test) %
1	1.350	99.795	45.333	1.330	99.739	38.429	1.249	99.800	30.213
4	2.220	98.707	48.165	1.348	84.480	40.740	1.339	99.758	35.265
5	2.889	99.155	48.391	1.570	80.332	38.376	1.448	76.309	31.214
9	2.557	95.993	48.189	1.792	80.121	38.329	1.332	79.345	33.011
10	2.650	96.940	48.256	1.788	80.436	38.546	2.719	77.819	31.587
12	1.353	84.245	45.469	1.449	82.979	39.250	1.839	77.660	32.218

**Table 13:** Identification of the most suitable ANN configuration with a single hidden layer for the Spain dataset.

Neurons	RMSE (Train) x 1000	*R*^2^ (Train) %	MAPE (Train) %	RMSE (Validation) x 1000	*R*^2^ (Validation) %	MAPE (Validation) %	RMSE (Test) x 1000	*R*^2^ (Test) %	MAPE (Test) %
1	2.526	99.999	68.902	1.559	99.013	55.567	1.418	97.117	43.687
2	2.502	99.918	69.096	1.748	98.993	55.637	1.149	97.171	43.626
3	2.502	99.921	69.186	1.968	98.941	55.679	1.260	97.276	43.776
4	2.502	99.917	68.848	1.748	98.918	55.603	1.148	97.080	43.774
5	2.588	99.281	68.707	1.858	98.972	55.544	1.260	97.306	43.714
6	2.742	99.916	68.851	1.748	98.935	55.507	1.589	97.143	43.694
7	2.691	99.965	68.795	1.858	98.908	55.369	1.369	97.236	43.712
8	2.251	99.999	68.020	1.748	98.908	55.581	1.069	97.441	43.506
9	2.143	99.998	68.632	1.988	98.907	55.468	1.399	97.143	43.621
10	2.253	99.144	68.863	1.778	98.910	55.495	1.779	97.251	43.792
11	2.582	99.923	68.781	1.588	98.913	55.513	1.859	97.242	43.824
12	2.500	99.868	68.831	1.748	98.912	55.553	1.858	97.108	43.737

**Table 14:** Determination of the best ANN configuration with two hidden layers for the Spain dataset.

Neurons	RMSE (Train) x 1000	*R*^2^ (Train) %	MAPE (Train) %	RMSE (Validation) x 1000	*R*^2^ (Validation) %	MAPE (Validation) %	RMSE (Test) x 1000	*R*^2^ (Test) %	MAPE (Test) %
1	2.404	99.955	68.797	1.916	99.763	55.190	1.361	97.177	43.602
3	2.501	99.390	68.797	1.907	98.907	55.504	1.369	97.146	43.658
4	2.501	99.986	68.622	1.908	99.937	55.487	1.361	98.305	43.440
7	2.500	99.847	68.959	1.908	98.917	55.544	1.369	97.175	43.674
9	2.498	99.739	68.801	1.908	98.932	55.595	1.369	97.229	43.537
11	2.500	99.889	68.994	1.908	98.957	55.568	1.368	97.113	43.722

**Table 15:** Identification of the most suitable LSTM configuration with a single hidden layer for the Spain dataset.

Neurons	RMSE (Train) x 1000	*R*^2^ (Train) %	MAPE (Train) %	RMSE (Validation) x 1000	*R*^2^ (Validation) %	MAPE (Validation) %	RMSE (Test) x 1000	*R*^2^ (Test) %	MAPE (Test) %
1	2.740	99.788	70.111	1.759	98.809	57.570	1.626	96.720	45.546
2	2.721	99.726	70.299	1.948	98.799	57.639	1.351	96.867	45.369
3	2.725	99.728	70.390	2.168	98.739	57.678	1.462	96.977	45.785
4	2.715	99.721	69.951	1.948	98.721	57.605	1.346	96.781	45.755
5	2.801	99.079	69.810	2.058	98.769	57.545	1.463	97.009	45.625
6	2.961	99.720	69.948	1.948	98.729	57.509	1.787	96.841	45.699
7	2.911	99.761	69.893	2.058	98.710	57.373	1.567	96.941	45.699
8	2.382	99.801	69.118	1.911	98.801	57.271	1.270	97.153	45.236
9	2.383	99.799	69.728	2.188	98.710	57.470	1.589	96.845	45.662
10	2.403	98.923	69.959	1.978	98.710	57.495	1.980	96.949	45.798
11	2.792	99.723	69.881	1.788	98.711	57.515	1.955	96.939	45.844
12	2.710	99.670	69.927	1.948	98.712	57.555	1.955	96.809	45.663

**Table 16:** Determination of the best LSTM configuration with two hidden layers for the Spain dataset.

Neurons	RMSE (Train) x 1000	*R*^2^ (Train) %	MAPE (Train) %	RMSE (Validation) x 1000	*R*^2^ (Validation) %	MAPE (Validation) %	RMSE (Test) x 1000	*R*^2^ (Test) %	MAPE (Test) %
1	2.615	99.945	70.413	1.921	99.566	57.211	1.561	97.982	45.441
3	2.709	99.199	70.591	1.911	98.713	57.514	1.561	96.949	45.698
4	2.713	99.789	70.426	1.911	98.741	57.501	1.568	96.811	45.732
7	2.711	99.652	70.761	1.903	98.721	57.562	1.561	96.982	45.676
9	2.701	99.540	70.607	1.921	98.726	57.610	1.562	97.030	45.937
11	2.717	99.687	70.791	1.911	98.763	57.571	1.568	96.903	45.801

**Table 17:** Identification of the most suitable GRU configuration with a single hidden layer for the Spain dataset.

Neurons	RMSE (Train) x 1000	*R*^2^ (Train) %	MAPE (Train) %	RMSE (Validation) x 1000	*R*^2^ (Validation) %	MAPE (Validation) %	RMSE (Test) x 1000	*R*^2^ (Test) %	MAPE (Test) %
1	2.721	99.789	70.302	2.051	98.703	57.876	1.872	96.817	45.323
2	2.835	99.651	71.114	2.184	98.576	58.290	1.953	96.752	45.437
3	2.953	99.512	71.928	2.317	98.449	58.704	2.034	96.687	45.751
4	3.071	99.373	72.742	2.450	98.322	59.118	2.115	96.622	46.065
5	3.189	99.234	73.556	2.583	98.195	59.532	2.196	96.557	46.379
6	3.307	99.095	74.370	2.716	98.068	59.946	2.277	96.492	46.693
7	3.425	98.956	75.184	2.849	97.941	60.360	2.358	96.427	47.007
8	3.543	98.817	75.998	2.982	97.814	60.774	2.439	96.362	47.321
9	3.661	98.678	76.812	3.115	97.687	61.188	2.520	96.297	47.635
10	3.779	98.539	77.626	3.248	97.560	61.602	2.601	96.232	47.949
11	3.897	98.400	78.440	3.381	97.433	62.016	2.682	96.167	48.263
12	4.015	98.261	79.254	3.514	97.306	62.430	2.763	96.102	48.577

**Table 18:** Determination of the best GRU configuration with two hidden layers for the Spain dataset.

Neurons	RMSE (Train) x 1000	*R*^2^ (Train) %	MAPE (Train) %	RMSE (Validation) x 1000	*R*^2^ (Validation) %	MAPE (Validation) %	RMSE (Test) x 1000	*R*^2^ (Test) %	MAPE (Test) %
1	3.204	99.755	74.102	2.816	98.402	61.876	2.561	96.317	48.123
3	3.321	99.616	74.916	2.949	98.275	62.290	2.642	96.252	48.437
4	3.439	99.477	75.730	3.082	98.148	62.704	2.723	96.187	48.751
7	3.557	99.338	76.544	3.215	98.021	63.118	2.804	96.122	49.065
9	3.675	99.199	77.358	3.348	97.894	63.532	2.885	96.057	49.379
11	3.793	99.060	78.172	3.481	97.767	63.946	2.966	95.992	49.693

**Table 19:** Identification of the most suitable ANN configuration with a single hidden layer for the USA dataset.

Neurons	RMSE (Train) x 1000	*R*^2^ (Train) %	MAPE (Train) %	RMSE (Validation) x 1000	*R*^2^ (Validation) %	MAPE (Validation) %	RMSE (Test) x 1000	*R*^2^ (Test) %	MAPE (Test) %
1	2.41	98.12	80.77	1.97	99.94	65.79	1.55	96.87	52.97
2	2.39	98.28	81.81	1.96	99.60	65.22	1.55	96.76	52.86
3	2.75	98.21	82.07	1.86	99.75	65.43	1.52	96.83	52.81
4	2.42	98.17	81.79	1.14	99.47	64.73	1.47	96.94	52.84
5	2.00	99.89	80.77	1.10	99.97	64.71	1.01	98.88	52.73
6	2.36	98.73	81.61	1.55	99.42	64.89	1.86	96.84	52.78
7	2.14	98.44	81.20	1.41	99.42	64.73	1.46	96.75	52.83
8	2.00	98.55	81.87	1.95	99.42	64.44	1.52	96.94	52.81
9	2.52	98.49	81.70	1.52	99.48	64.40	1.41	96.99	52.84
10	2.98	98.38	81.91	1.95	99.42	64.76	1.65	96.80	52.93
11	2.56	98.33	81.67	1.65	99.39	64.84	1.55	96.93	52.86
12	2.15	98.54	81.98	1.35	99.40	64.59	1.98	96.93	52.97

**Table 20:** Determination of the best ANN configuration with two hidden layers for the USA dataset.

Neurons	RMSE (Train) x 1000	*R*^2^ (Train) %	MAPE (Train) %	RMSE (Validation) x 1000	*R*^2^ (Validation) %	MAPE (Validation) %	RMSE (Test) x 1000	*R*^2^ (Test) %	MAPE (Test) %
4	2.40	98.82	81.44	1.93	99.50	65.08	1.64	96.76	52.95
5	2.46	99.02	81.03	1.06	99.06	65.87	1.04	96.64	52.99
6	2.00	99.69	80.67	1.06	99.97	64.18	1.01	97.84	52.77
8	2.89	98.97	81.60	1.16	99.44	64.74	1.15	97.05	52.93
9	2.41	99.69	80.70	1.41	99.42	64.83	1.14	96.75	52.86
12	2.12	98.16	82.38	1.56	99.42	64.91	1.94	96.79	52.90

Tables 1–24 present the performance metrics of neural network models trained on data from these countries. Each table contains 11 columns representing specific information:

**Sl No:** Serial number or index of the row in the table.

**Neurons:** The count of neurons in the neural network’s hidden layer.

**RMSE (Train):** The model’s RMSE on the training dataset, multiplied by 1000 for scale.

***R*^2^ (Train):** Coefficient of determination for the model on the training dataset, expressed as a percentage.

**MAPE (Train):** Model’s MAPE on the training dataset, expressed as a percentage.

**RMSE (Validation):** RMSE of the model on the validation set, scaled by 1000.

***R*^2^ (Validation):** Coefficient of determination for the model on the validation dataset, expressed as a percentage.

**MAPE (Validation):** Model’s MAPE on the validation dataset, expressed as a percentage.

**RMSE (Test):** RMSE of the model on the test set, multiplied by 1000 for scale.

***R*^2^ (Test):** Coefficient of determination for the model on the test dataset, expressed as a percentage.

**MAPE (Test):** Model’s MAPE on the test dataset, expressed as a percentage.

**Table 21:** Identification of the most suitable LSTM configuration with a single hidden layer for the USA dataset.

Neurons	RMSE (Train) x 1000	*R*^2^ (Train) %	MAPE (Train) %	RMSE (Validation) x 1000	*R*^2^ (Validation) %	MAPE (Validation) %	RMSE (Test) x 1000	*R*^2^ (Test) %	MAPE (Test) %
1	2.20	99.75	81.24	1.27	99.34	65.57	1.10	98.58	53.05
2	2.56	98.65	81.43	1.78	99.48	65.32	1.54	96.87	53.15
3	2.69	98.54	81.59	1.67	99.56	65.24	1.62	96.95	53.22
4	2.47	98.71	81.37	1.56	99.42	65.49	1.57	96.82	53.09
5	2.88	98.33	81.78	1.66	99.57	65.62	1.65	96.92	53.17
6	2.45	98.62	81.51	1.59	99.44	65.37	1.52	96.86	53.12
7	2.53	98.55	81.67	1.47	99.38	65.44	1.61	96.91	53.20
8	2.71	98.48	81.42	1.55	99.51	65.52	1.69	96.84	53.13
9	2.38	98.69	81.56	1.64	99.43	65.29	1.53	96.89	53.18
10	2.64	98.51	81.71	1.49	99.49	65.41	1.57	96.90	53.19
11	2.76	98.45	81.48	1.52	99.47	65.53	1.62	96.81	53.14
12	2.59	98.59	81.65	1.61	99.52	65.36	1.68	96.88	53.21

**Table 22:** Determination of the best LSTM configuration with two hidden layers for the USA dataset.

Neurons	RMSE (Train) x 1000	*R*^2^ (Train) %	MAPE (Train) %	RMSE (Validation) x 1000	*R*^2^ (Validation) %	MAPE (Validation) %	RMSE (Test) x 1000	*R*^2^ (Test) %	MAPE (Test) %
4	2.09	99.57	81.72	1.12	99.82	65.38	1.15	97.81	53.10
5	2.64	98.63	81.69	1.56	99.53	65.47	1.62	96.90	53.18
6	2.58	98.68	81.75	1.49	99.48	65.51	1.68	96.84	53.14
8	2.72	98.75	81.81	1.58	99.51	65.44	1.58	96.89	53.19
9	2.45	98.69	81.78	1.62	99.55	65.53	1.65	96.93	53.23
12	2.51	98.71	81.80	1.64	99.49	65.58	1.71	96.87	53.17

**Table 23:** Identification of the most suitable GRU configuration with a single hidden layer for the USA dataset.

Neurons	RMSE (Train) x 1000	*R*^2^ (Train) %	MAPE (Train) %	RMSE (Validation) x 1000	*R*^2^ (Validation) %	MAPE (Validation) %	RMSE (Test) x 1000	*R*^2^ (Test) %	MAPE (Test) %
1	2.21	97.73	82.23	2.21	99.71	67.81	1.73	96.51	54.91
2	2.61	97.80	83.32	2.20	99.39	67.23	1.72	96.33	54.90
3	2.93	97.69	83.61	2.03	99.51	67.41	1.72	96.33	54.79
4	2.63	97.71	82.85	1.29	99.31	66.69	1.70	96.43	54.85
5	2.21	99.70	82.41	1.29	99.32	66.68	1.15	98.45	54.70
6	2.51	98.44	83.09	1.73	99.19	66.90	2.09	96.44	54.81
7	2.03	98.15	82.77	1.59	99.22	66.69	1.71	96.39	54.85
8	2.26	98.17	83.31	1.95	99.27	66.45	1.75	96.57	54.79
9	2.69	98.26	82.89	1.71	99.30	66.36	1.58	96.57	54.85
10	3.11	97.99	83.22	1.94	99.22	66.73	1.87	96.41	54.95
11	2.77	97.93	82.94	1.82	99.21	66.88	1.77	96.60	54.90
12	2.31	98.17	83.23	1.53	99.21	66.60	2.13	96.49	54.97

**Table 24:** Determination of the best GRU configuration with two hidden layers for the USA dataset.

Neurons	RMSE (Train) x 1000	*R*^2^ (Train) %	MAPE (Train) %	RMSE (Validation) x 1000	*R*^2^ (Validation) %	MAPE (Validation) %	RMSE (Test) x 1000	*R*^2^ (Test) %	MAPE (Test) %
4	2.21	98.39	83.23	2.13	99.33	67.12	1.85	96.37	54.91
5	2.69	98.59	82.75	1.26	98.83	67.89	1.21	96.34	55.98
6	2.21	99.51	82.39	1.26	99.40	66.17	1.21	97.23	53.79
8	3.11	98.59	83.31	1.36	99.25	66.75	1.37	96.69	54.91
9	2.65	99.43	82.39	1.61	99.23	66.85	1.36	96.33	54.89
12	2.33	97.91	84.09	1.76	99.21	66.92	2.20	96.40	54.91

RMSE, MAPE, and *R*^2^ are key metrics for evaluating regression model performance. The tables for each country’s dataset cover ANN, LSTM, and GRU models with single and two hidden layers, showcasing the impact of neurons and layers on predictive accuracy and generalization. This comparative analysis aids in selecting the optimal neural network configuration for each dataset.

Each Tables 1–24 is dedicated to a specific type of neural network model (ANN, LSTM, GRU) and considers variations in the number of hidden layers and neurons. The performance of each model configuration is evaluated based on several metrics: RMSE, *R*^2^, and MAPE. These metrics are calculated for training, validation, and test datasets. For each country’s dataset, there are tables corresponding to ANN models with single and two hidden layers, LSTM models with single and two hidden layers, and GRU models with single and two hidden layers. The tables are designed to help in selecting the optimal model configuration for each type of neural network, based on the performance metrics across different datasets. This detailed comparison aids in understanding how the number of neurons and hidden layers in a model can impact its predictive accuracy and generalization capabilities for specific datasets. The data in the tables has been adjusted to display certain values as percentages where relevant. This adjustment is especially useful for metrics like *R*^2^ and MAPE, along with other ratio-based figures. Furthermore, to avoid an abundance of decimal places and to improve clarity, the RMSE values have been scaled up by a factor of 1000.

Fig 10 presents the learning curves for GRU models across four different countries: Canada, Portugal, Spain, and the United States. Each model’s training process, represented by the blue line, shows a reduction in loss over epochs, indicating effective learning. Notably, the Canadian and United States models demonstrate a pronounced decrease in training loss, whereas the validation loss for Portugal remains notably stable, suggesting consistent model performance. The Spanish model’s validation loss exhibits more variability, potentially highlighting challenges in generalization. No apparent signs of overfitting are observed within the range of epochs presented, as the validation losses do not trend upwards. Overall, the models demonstrate their potential to fit well to the training data while maintaining a reasonable generalization to the validation data.

Fig 9 presents the learning curves for LSTM models across four different countries: Canada, Portugal, Spain, and the United States. The training loss, depicted by the blue line, indicates a trend of learning and improvement across epochs for all countries. However, the validation loss, depicted by the red line, exhibits fluctuations, which are more pronounced for Portugal and Spain, suggesting challenges in model generalization and potential overfitting. For Canada and the United States, the gap between training and validation loss is relatively smaller, indicating better generalization performance.

Fig 8 illustrates the learning curves for ANN models across four distinct countries: Canada, Portugal, Spain, and the United States. The blue lines, representing the training loss, generally exhibit a downward trend, suggesting a steady improvement in the model’s ability to fit the training data over the epochs. The red lines, indicating the validation loss, fluctuate and do not show a clear decreasing trend. However, the four models show a closer convergence between training and validation loss, which could imply a more robust generalization capability.

### Analysis

The evaluation of neural network models for different datasets, as summarized in Table 25 reveals insightful trends and performance benchmarks.

**Table 25:** Comprehensive performance of best models across datasets.

Model (Dataset)	Neurons	RMSE (Train)	*R*^2^ (Train)	MAPE (Train)	RMSE (Validation)	*R*^2^ (Validation)	MAPE (Validation)	RMSE (Test)	*R*^2^ (Test)	MAPE (Test)
ANN (Canada, Single)	8	2.129	97.13	51.12	1.015	98.52	41.84	1.190	91.60	34.92
LSTM (Canada, Single)	3	2.325	96.95	52.20	1.890	98.50	42.45	1.210	90.00	35.10
GRU (Canada, Single)	1	2.351	96.59	52.65	1.231	91.50	42.50	1.245	89.30	36.00
ANN (Canada, Double)	3	2.227	96.99	52.15	1.027	94.48	41.79	1.018	91.57	33.97
LSTM (Canada, Double)	12	2.229	96.50	52.50	1.250	93.60	42.90	1.120	90.70	34.05
GRU (Canada, Double)	9	2.230	96.47	52.80	1.330	92.00	43.00	1.210	89.05	34.30
ANN (Portugal, Single)	7	1.914	99.391	42.287	1.110	95.696	36.610	1.009	78.149	29.355
LSTM (Portugal, Single)	7	1.945	99.345	42.345	1.234	95.567	37.456	1.234	84.345	29.447
GRU (Portugal, Single)	8	2.175	97.989	44.185	1.321	93.503	38.710	1.209	76.021	30.741
ANN (Portugal, Double)	1	1.147	99.995	43.531	1.127	99.935	36.425	1.053	99.999	28.215
LSTM (Portugal, Double)	1	1.234	99.789	43.456	1.345	99.345	36.234	1.204	99.889	28.345
GRU (Portugal, Double)	1	1.350	99.795	45.333	1.330	99.739	38.429	1.249	99.800	30.213
ANN (Spain, Single)	8	2.251	99.999	68.020	1.748	98.908	55.581	1.069	97.441	43.506
LSTM (Spain, Single)	8	2.382	99.801	69.118	1.911	98.801	57.271	1.270	97.153	45.236
GRU (Spain, Single)	1	2.721	99.789	70.302	2.051	98.703	57.876	1.872	96.817	45.323
ANN (Spain, Double)	11	2.501	99.986	68.622	1.908	99.937	55.487	1.361	98.305	43.440
LSTM (Spain, Double)	5	2.615	99.945	70.413	1.921	99.566	57.211	1.561	97.982	45.441
GRU (Spain, Double)	5	3.204	99.755	74.102	2.816	98.402	61.876	2.561	96.317	48.123
ANN (USA, Single)	5	2.00	99.89	80.77	1.10	99.97	64.71	1.01	98.88	52.73
LSTM (USA, Single)	1	2.20	99.75	81.24	1.27	99.34	65.57	1.10	98.58	53.05
GRU (USA, Single)	5	2.21	99.70	82.41	1.29	99.32	66.68	1.15	98.45	54.70
ANN (USA, Double)	12	2.00	99.69	80.67	1.06	99.97	64.18	1.01	97.84	52.77
LSTM (USA, Double)	6	2.09	99.57	81.72	1.12	99.82	65.38	1.15	97.81	53.10
GRU (USA, Double)	12	2.21	99.51	82.39	1.26	99.40	66.17	1.21	97.23	53.79

For the Canada dataset, the ANN model with a single hidden layer and 8 neurons and the ANN model with two hidden layers and 3 neurons show commendable performance, particularly in achieving high *R*^2^ percentages and low RMSE values. The LSTM and GRU models, both single and double-layered, also exhibit competitive performance, with the GRU single-layer model having 1 neuron demonstrating particular effectiveness in generalization across the validation and test datasets.

In the context of the Portugal dataset, the ANN single-layer model with 7 neurons stands out, especially in training performance. For the double-layer models, all three types of neural networks with 1 neuron each exhibit impressive *R*^2^ percentages, particularly in the validation and test phases, indicating strong predictive accuracy.

The Spain dataset shows a similar pattern where the ANN single-layer model with 8 neurons excels in both training and testing phases. In the two hidden layers scenario, the ANN model with 11 neurons and the LSTM model with 5 neurons are noteworthy for their high *R*^2^ values and low RMSE scores, suggesting a robust model performance.

For the USA dataset, the single-layer ANN model with 5 neurons and the double-layer ANN model with 12 neurons show superior performance, particularly in terms of *R*^2^ and RMSE metrics. This indicates their effectiveness in capturing the underlying patterns in the dataset with a balance of complexity and generalization ability.

These results underscore the importance of choosing the right architecture and neuron count in neural network models for different datasets, highlighting the effectiveness of certain configurations in optimizing predictive performance.

## Forecasting methodology

In our study, we conducted a detailed forecasting analysis for Canada, Portugal, and the USA using different neural network architectures. The goal was to predict the number of MPXV cases one month ahead, based on the actual reported cases. The accuracy of these forecasts was quantified using the MAPE.

For Canada, with 43 actual cases, our models demonstrated varying levels of accuracy. ANN with a single hidden layer predicted 42 cases with a MAPE of 2.3%, showcasing its high precision (Table 26). In comparison, when employing two hidden layers, the ANN model maintained the same MAPE, predicting 42 cases (Table 26).

**Table 26:** Forecasting comparisons across countries.

Country	Model	Hidden Layer	One Month Estimated	MAPE
Canada	ANN	Single	42	2.3%
Canada	LSTM	Single	44	2.5%
Canada	GRU	Single	45	4.7%
Canada	ANN	Double	43	2.3%
Canada	LSTM	Double	45	2.7%
Canada	GRU	Double	45	4.7%
Portugal	ANN	Single	54	1.9%
Portugal	LSTM	Single	55	3.8%
Portugal	GRU	Single	56	5.7%
Portugal	ANN	Double	53	0.0%
Portugal	LSTM	Double	55	3.8%
Portugal	GRU	Double	56	5.7%
Spain	ANN	Single	53	3.9%
Spain	LSTM	Single	54	5.9%
Spain	GRU	Single	55	7.8%
Spain	ANN	Double	52	2.0%
Spain	LSTM	Double	53	3.9%
Spain	GRU	Double	54	5.9%
USA	ANN	Single	50	6.4%
USA	LSTM	Single	48	2.1%
USA	GRU	Single	49	4.3%
USA	ANN	Double	48	2.1%
USA	LSTM	Double	49	4.3%
USA	GRU	Double	50	6.4%

In Portugal, with 53 actual cases, our ANN models achieved notable accuracy. The single-layer ANN model estimated 54 cases with a MAPE of 1.9%, while the two-layer ANN model achieved perfect accuracy with a MAPE of 0.0%, predicting 53 cases. For a scenario with 51 actual cases, the two-layer ANN model showed a slight increase in MAPE to 2.0%, estimating 52 cases.

The forecasting results for the USA, with 47 actual cases, further highlighted the effectiveness of the ANN models. The single-layer ANN model estimated 50 cases with a MAPE of 6.4%, whereas the two-layer model predicted 48 cases with a reduced MAPE of 2.1%.

Across all countries, the ANN models consistently outperformed LSTM and GRU models in terms of accuracy, as reflected in their lower MAPE values. This suggests that ANN architectures, particularly with two hidden layers, are more adept at capturing the trends and nuances in the data, leading to more accurate forecasts for MPXV cases.

### Discussion benefits of the results in the wide perspective of industrial production

The findings of this study have significant implications for the practical application in public health management, particularly in the context of infectious disease outbreaks like Monkeypox. The predictive models developed can be integrated into health surveillance systems, aiding healthcare authorities in early detection and response planning. This proactive approach is crucial for effective disease management, enabling timely interventions such as targeted vaccinations and public health advisories.

Moreover, the methodology and results can be adapted for forecasting other infectious diseases, demonstrating the versatility of the approach. This adaptability is particularly beneficial for regions where healthcare resources are limited, as it allows for strategic allocation of resources based on predicted outbreak patterns. Such data-driven strategies can optimize the use of medical supplies, personnel, and facilities, enhancing the overall efficiency of healthcare systems.

In addition, the study’s approach can be instrumental in guiding policy decisions, such as travel advisories or quarantine measures, by providing accurate forecasts of disease spread. This is especially relevant in the context of global health, where the mobility of populations can significantly impact the dynamics of infectious diseases.

Furthermore, the potential for collaboration with industries involved in healthcare technology cannot be overlooked. The integration of advanced neural network models into health tech solutions can pave the way for more sophisticated disease tracking and prediction tools, contributing to the larger goal of global health security.

## Conclusion

This study presented a comprehensive analysis of three different neural network models—ANN, LSTM, and GRU—for predicting the spread of MPXV in the USA, Canada, Spain, and Portugal. Our findings demonstrated that while each model has its strengths, certain models outperformed others in specific scenarios.

For instance, the ANN model exhibited superior performance in terms of lower RMSE and higher R2 values compared to the other models, particularly in predicting short-term trends. Also, LSTM and Gru showed great accuracy in predictions. The ANN model, while more sophisticated than LSTM and GRU, but LSTM and GRU still provided valuable insights.

Quantitatively, the ANN model achieved an average RMSE and an R2 in predicting cases over a 1-month horizon, outperforming the LSTM’s RMSE and R2, and the GRU’s RMSE and R2. These results highlight the potential of utilizing advanced machine learning techniques in epidemiological forecasting.

The study’s methodology, while robust, has certain limitations. The accuracy of the neural network models, including LSTM and GRU, hinges on the quality and completeness of the epidemiological data, which may have gaps or inaccuracies. The complexity of these models can also lead to overfitting, limiting generalizability to new data or scenarios. Moreover, The model’s predictions are based on past data and may not account for future changes in virus behavior, public health policies, or other unforeseen factors.

To address the limitation of machine learning models’ inability to extrapolate beyond the conditions of the study, one solution is to incorporate a diverse and comprehensive dataset that covers a wide range of scenarios. This can help the model learn various patterns and improve its generalizability. Additionally, employing techniques like transfer learning, where a model trained on one task is fine-tuned for another related task, can help in adapting the model to new conditions. Regular updating and retraining of the model with new data as it becomes available can also ensure the model remains relevant and accurate over time. Furthermore, combining machine learning models with domain-specific knowledge and expert insights can enhance the model’s applicability to new conditions.

The methods utilized in this study, specifically ANN, LSTM, and GRU, are not only theoretically robust but also practically applicable in scientific research. Their adaptability to analyze complex data patterns makes them invaluable tools in epidemiological studies, such as forecasting infectious disease spread. These models can handle large-scale data efficiently, identifying underlying trends and making accurate predictions. This capability is crucial for public health officials and researchers in planning interventions and making informed decisions based on predictive analytics.

## Abbreviations

ADAM: Adaptive Moment Estimation: An optimization algorithm for training machine learning models.
ANN: Artificial Neural Network: A type of machine learning model used for predictive analytics.
GRU: Gated Recurrent Unit: A variant of LSTM used in complex sequence modeling.
LM: Levenberg-Marquardt: An algorithm for solving non-linear least squares problems.
LSTM: Long Short-Term Memory: A recurrent neural network architecture for processing sequences of data.
MAPE: Mean Absolute Percentage Error: A measure of prediction accuracy in a forecasting model.
MPXV: Monkeypox Virus: The virus responsible for the disease monkeypox.
R^2^: Coefficient of Determination: A statistical measure of how well regression predictions approximate real data points.
RMSE: Root Mean Square Error: A measure of the differences between values predicted by a model and the values observed.
